# Supplementary material for: TSC2 regulates lysosome biogenesis via a non-canonical RAGC and TFEB-dependent mechanism
Source: Nat Commun. 2021 Jul 12;12:4245. doi: 10.1038/s41467-021-24499-6 (PMC8275687; doi:10.1038/s41467-021-24499-6)
Supplement: Supplementary file 1 — Supplementary Information [file 41467_2021_24499_MOESM1_ESM.pdf]

Supplementary information

TSC2 regulates lysosome biogenesis via a non-canonical RAGC and TFEB-dependent mechanism

Alesi et al.

Supplementary Figure 1

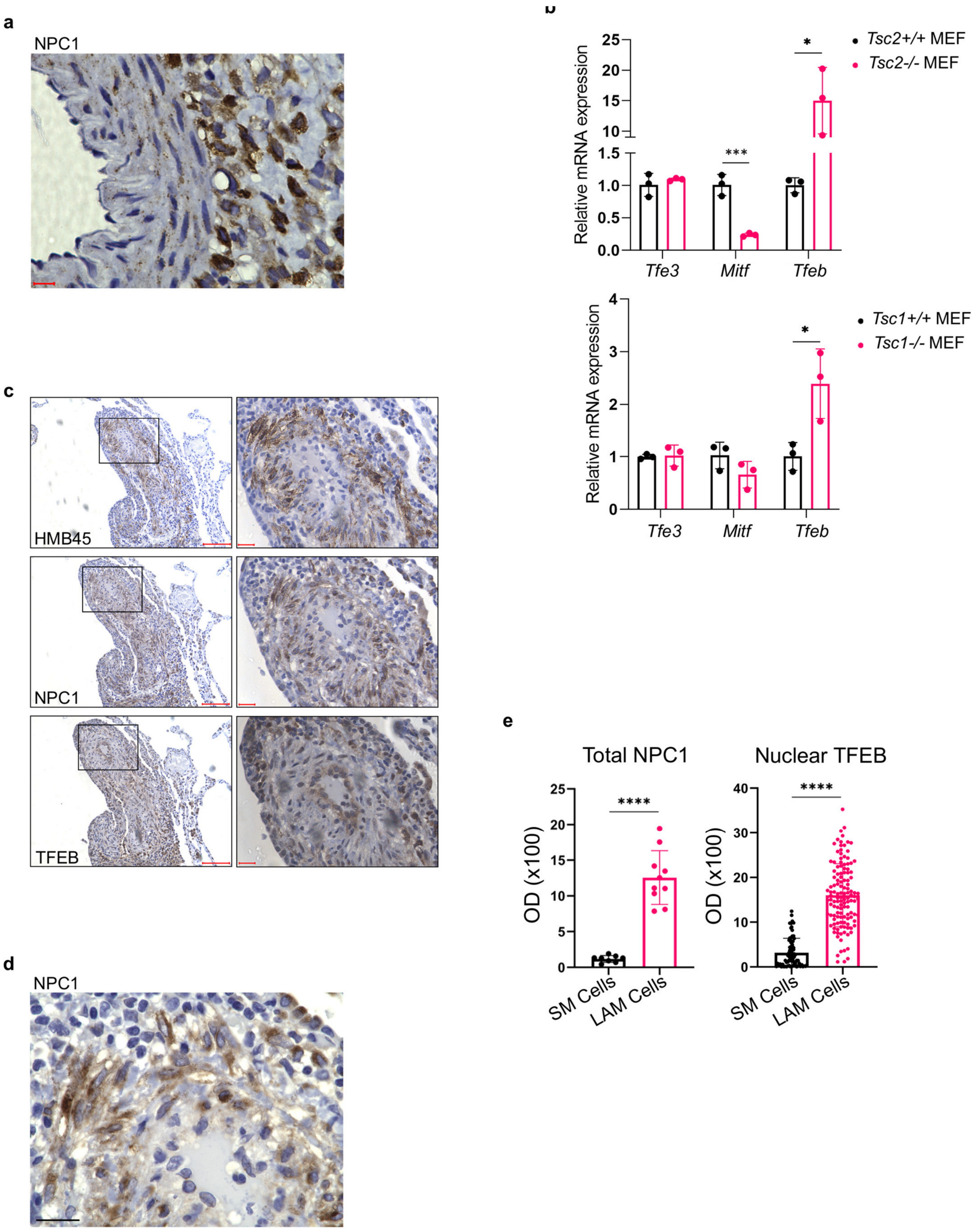

Supplementary Figure 1. Elevated expression of TFEB in *Tsc1*- and *Tsc2*-deficient MEFs and in LAM nodules

a, Immunohistochemistry for the lysosomal marker NPC1 in human renal angiomyolipoma at 100x magnification (n=3 patient samples). Scale bar= 10 µm. **b**, qRT-PCR analysis of *Tfe3, Mitf* and *Tfeb* in $Tsc1^{-/-}$ and $Tsc2^{-/-}$ MEFs compared to their wild-type counterparts (n=3 biological replicates). **c, d**, **e**, IHC analysis of NPC1 and TFEB expression in HMB-45 positive LAM nodules (left panels, scale bars= 100 µm; right panels, scale bars= 20 µm) (**c**), NPC1 staining at 100x magnification in LAM nodule. Scale bar= 20 µm (**d**). Optical density of total NPC1 and nuclear TFEB in LAM cells from HMB45 positive nodules was quantified using ImageJ (8 measurements for NPC1 in SM cells and 10 measurements for LAM cells; 76 measurements for TFEB in SM cells and 126 measurements in LAM cells in 6 HMB45 positive nodules from 2 different patient samples) (**e**). Graphs are presented as mean ± SD. Statistical analyses were performed using two-tailed Students t-test, *p<0.05, **p<0.01, ****p<0.0001. Source data are provided as a Source data file.

Supplementary Figure 2

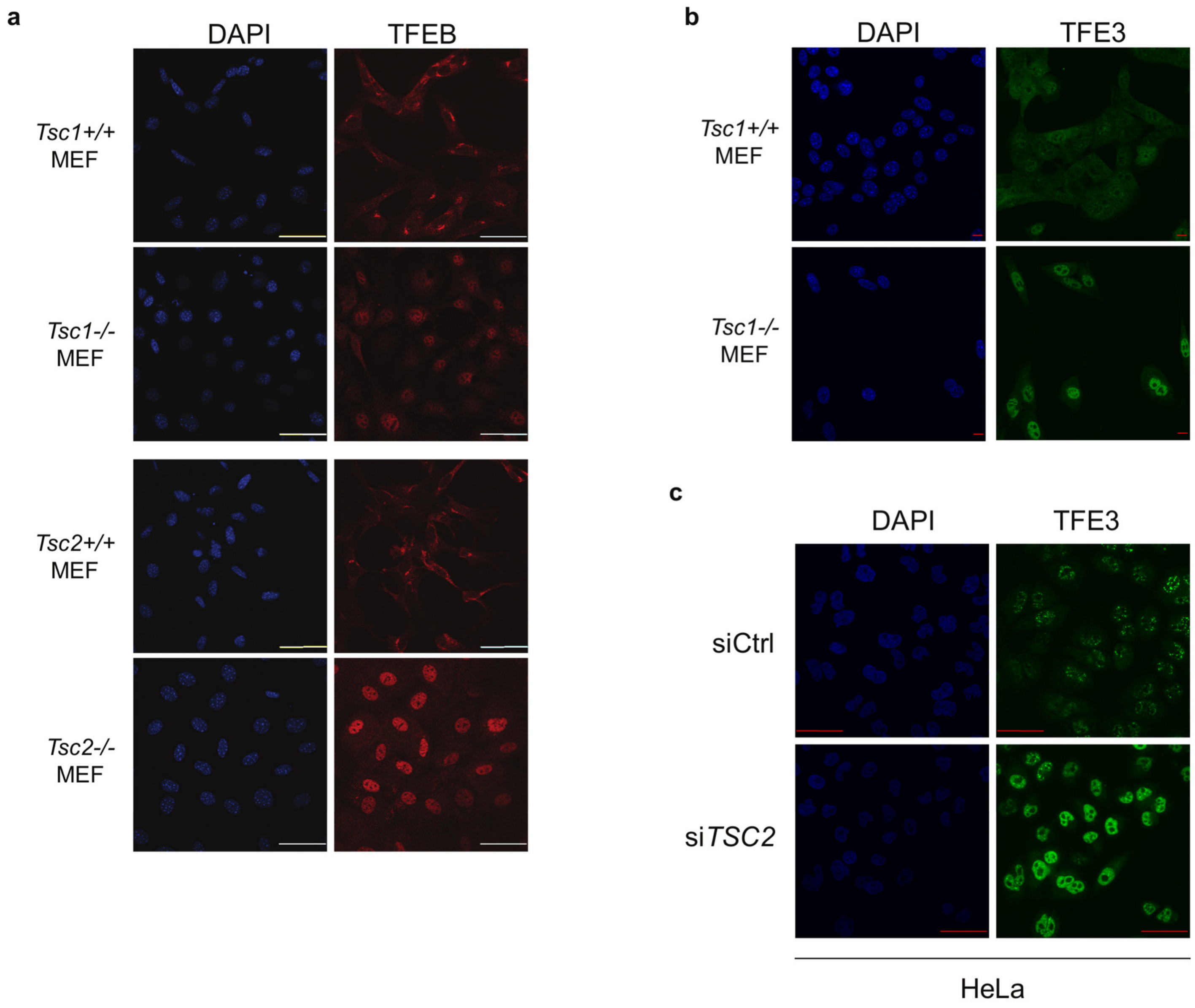

Supplementary Fig 2. TFEB and TFE3 predominantly localize to the nucleus in Tsc1/Tsc2-deficient MEFs and HeLa cells with TSC2 downregulation

a, Immunofluorescent analysis of TFEB localization in Tsc1- and Tsc2-deficient MEFs compared to their wild type counterparts (n=3 biological replicates per condition). b, c, Immunofluorescent analysis of TFE3 localization in Tsc1-deficient and Tsc1-expressing MEFs (b) and HeLa cells with siTSC2 for 72hr (c) (n=3 biological replicates per condition). Scale bars= 50 µm in a, 10 µm in b, =50µm in c.

Supplementary Figure 3

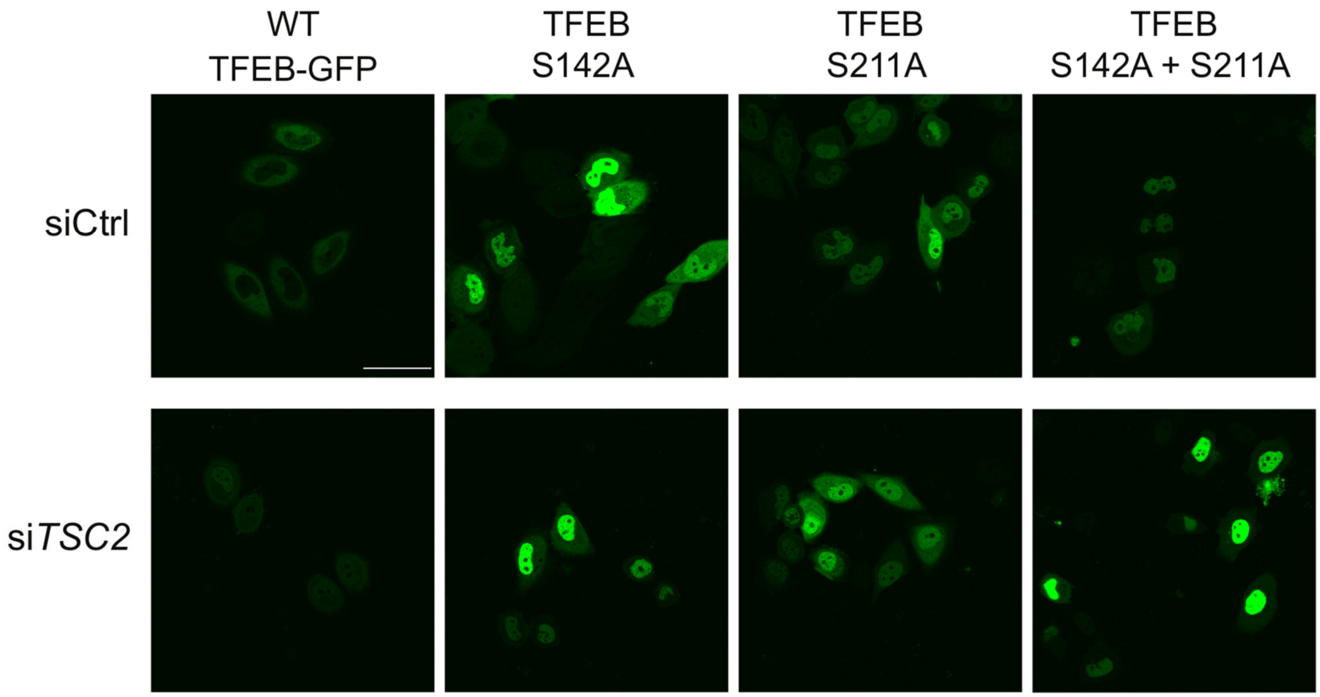

Supplementary Fig 3. ***TSC2*** **downregulation does not change the localization of mTORC1-resistant S142A and S211A mutants of TFEB**

HeLa cells with *Ctrl* or *TSC2* siRNA were transfected with indicated TFEB-GFP plasmids for 48hr and visualized with confocal live imaging (n=3 biological replicates per condition). Scale bars= 50 μm.

Supplementary Figure 4

a

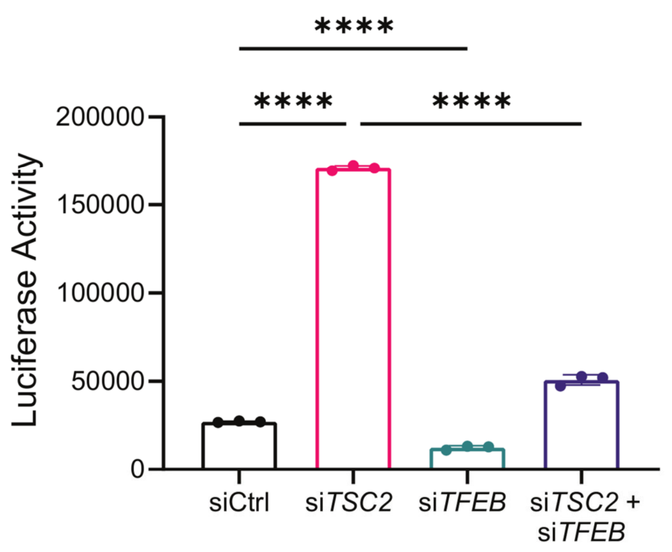

b

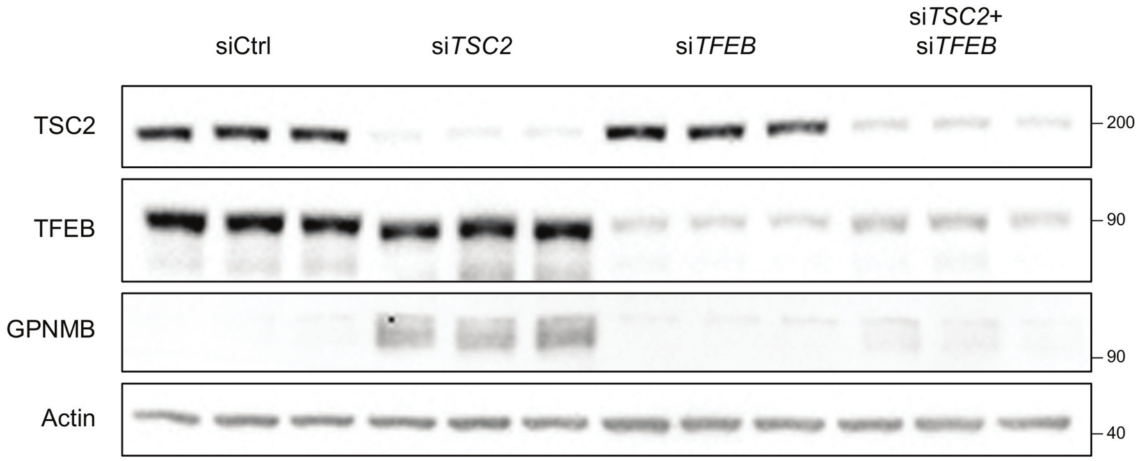

c

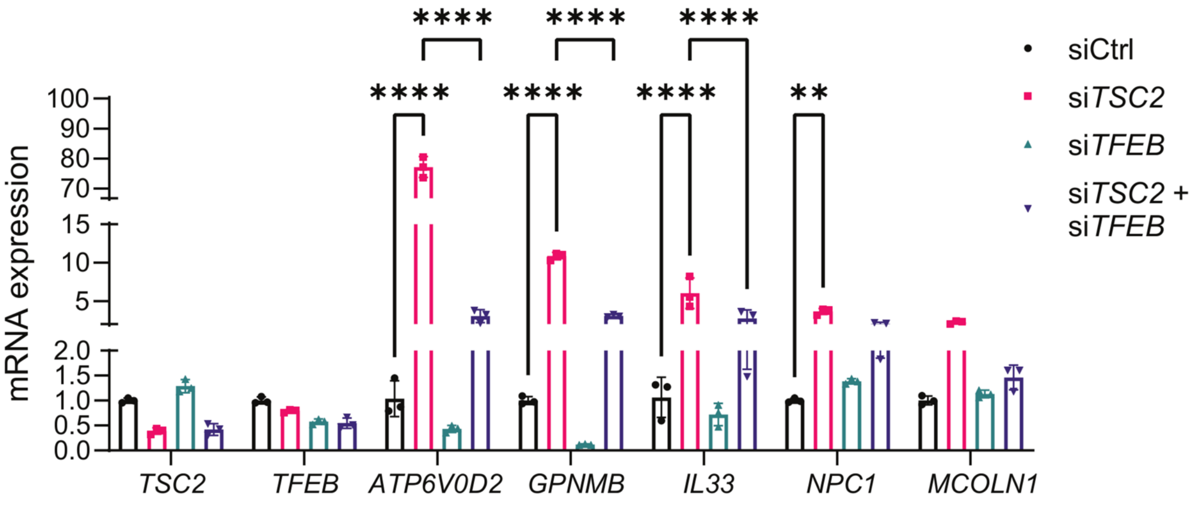

Supplementary Fig 4. Lysosomal gene expression and GPNMB reporter activity and protein expression are TFEB-dependent in TSC2-deficient cells

a, Luciferase activity of HeLa-TFEB-GFP stably expressing the GPNMB luciferase reporter and transfected with indicated siRNAs for 72 hr (n=3 biological replicates per condition). b, Immunoblot analysis of cells treated as in a with indicated antibodies (biological triplicates). c, qRT-PCR analysis of TFEB target genes in cells treated as in a (n=3 biological replicates per condition). Graphs are presented as mean ± SD. Statistical analyses were performed using two-tailed Students t-test, or one-way ANOVA if more than 2 groups, $**p<0.01$ $***p<0.001$, $****p<0.0001$. Source data are provided as a Source data file.

Supplementary Figure 5

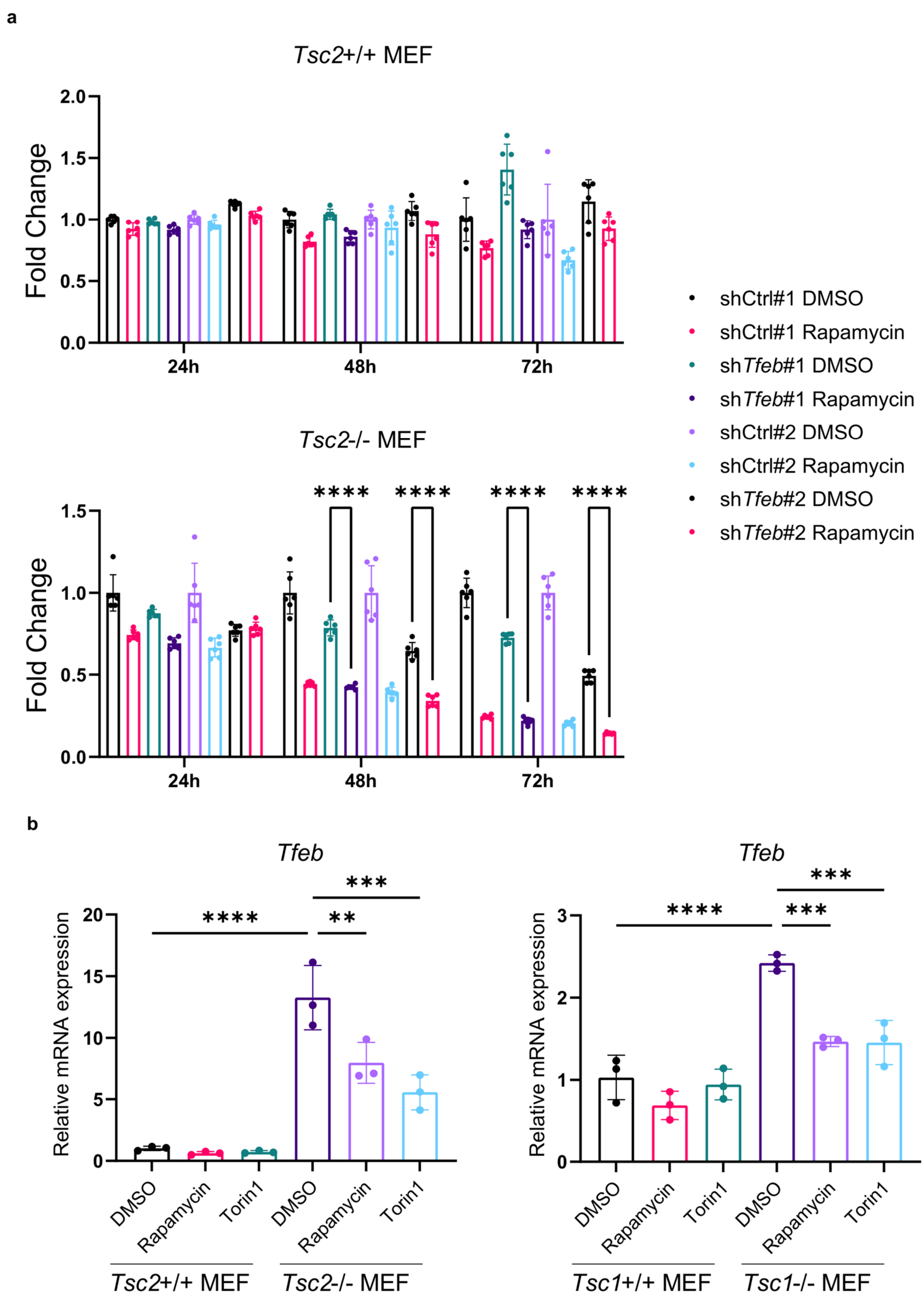

Supplementary Fig 5. Rapamycin and Torin1 decrease *Tfeb* mRNA levels in *Tsc1*- and *Tsc2*-deficient MEFs

a, Proliferation of *Tsc2*$^{+/+}$ and *Tsc2*$^{-/-}$ MEFs with control or *Tfeb* shRNA, treated with Dmso or Rapamycin (20nM) for indicated times and assessed by crystal violet staining (n=6 biological replicates each condition). **b**, qRT-PCR analysis of *Tfeb* expression in *Tsc1*$^{+/+,}$ *Tsc1*$^{-/-}$ MEFs, *Tsc2*$^{+/+}$ and *Tsc2*$^{-/-}$ MEFs treated with Dmso, Rapamycin (20nM) or Torin1 (250nM) for 24hr (n=3 biological replicates). Graphs are presented as mean ± SD. Statistical analyses were performed using two-tailed Students t-test, or one-way ANOVA if more than 2 groups, **p<0.01, ***p<0.001, ****p<0.0001. Source data are provided as a Source data file.

Supplementary Figure 6

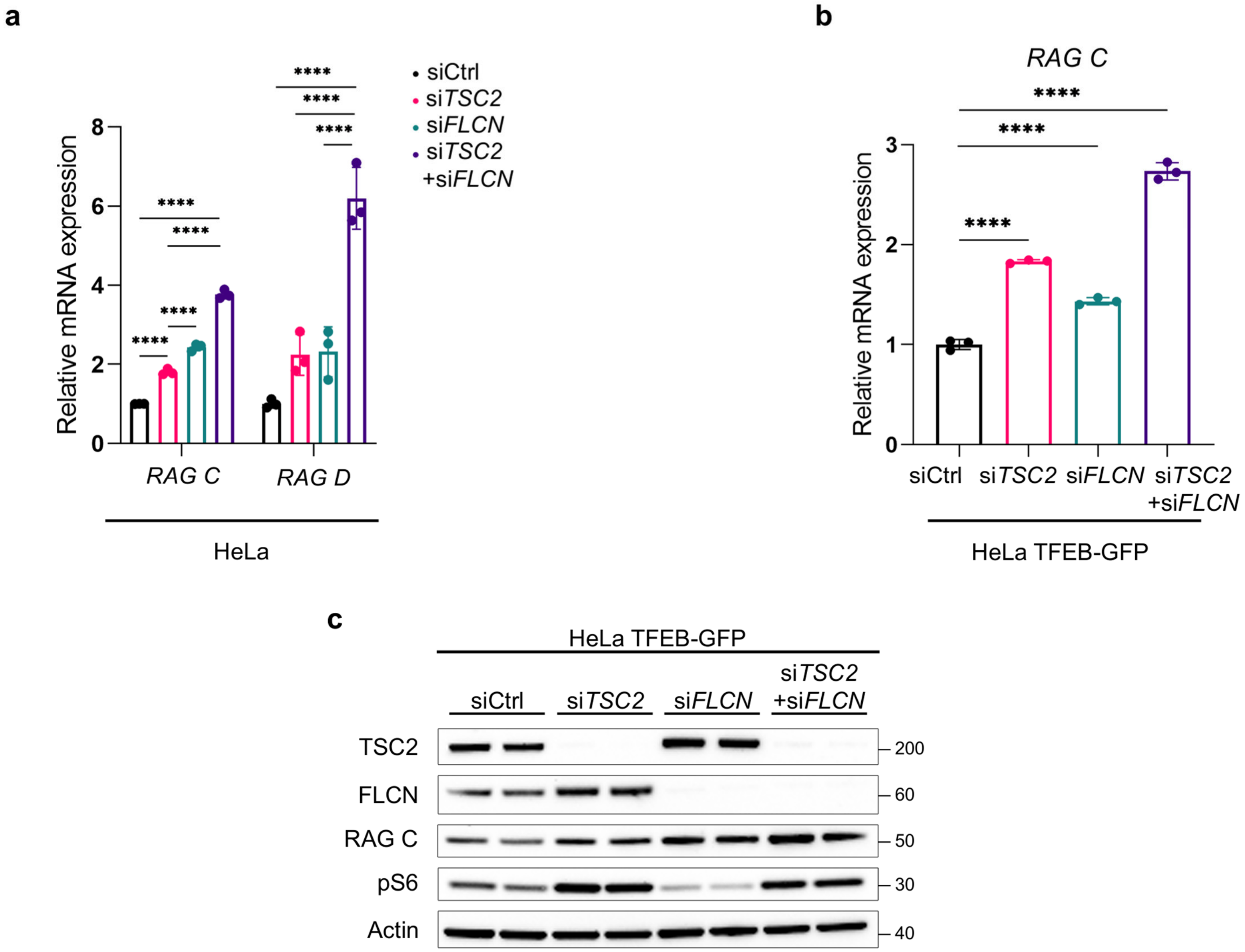

Supplementary Figure 6. Expression of RAGC and RAGD is increased in cells with downregulation of TSC2, FLCN or both

a, b, qRT-PCR analysis of RAGC and RAGD in HeLa cells, (n=3 biological replicates per condition) (a) and of RAGC in HeLa-TFEB-GFP (n=3 biological replicates per condition) (b) after transfection with indicated siRNAs for 72 hr. RAGD was not detectable in HeLa-TFEB-GFP cells. c, Immunoblot analysis of cells transfected as in b with indicated antibodies. Graphs are presented as mean ± SD. Statistical analyses were performed using one-way ANOVA, ****p<0.0001. Source data are provided as a Source data file.

Supplementary Figure 7

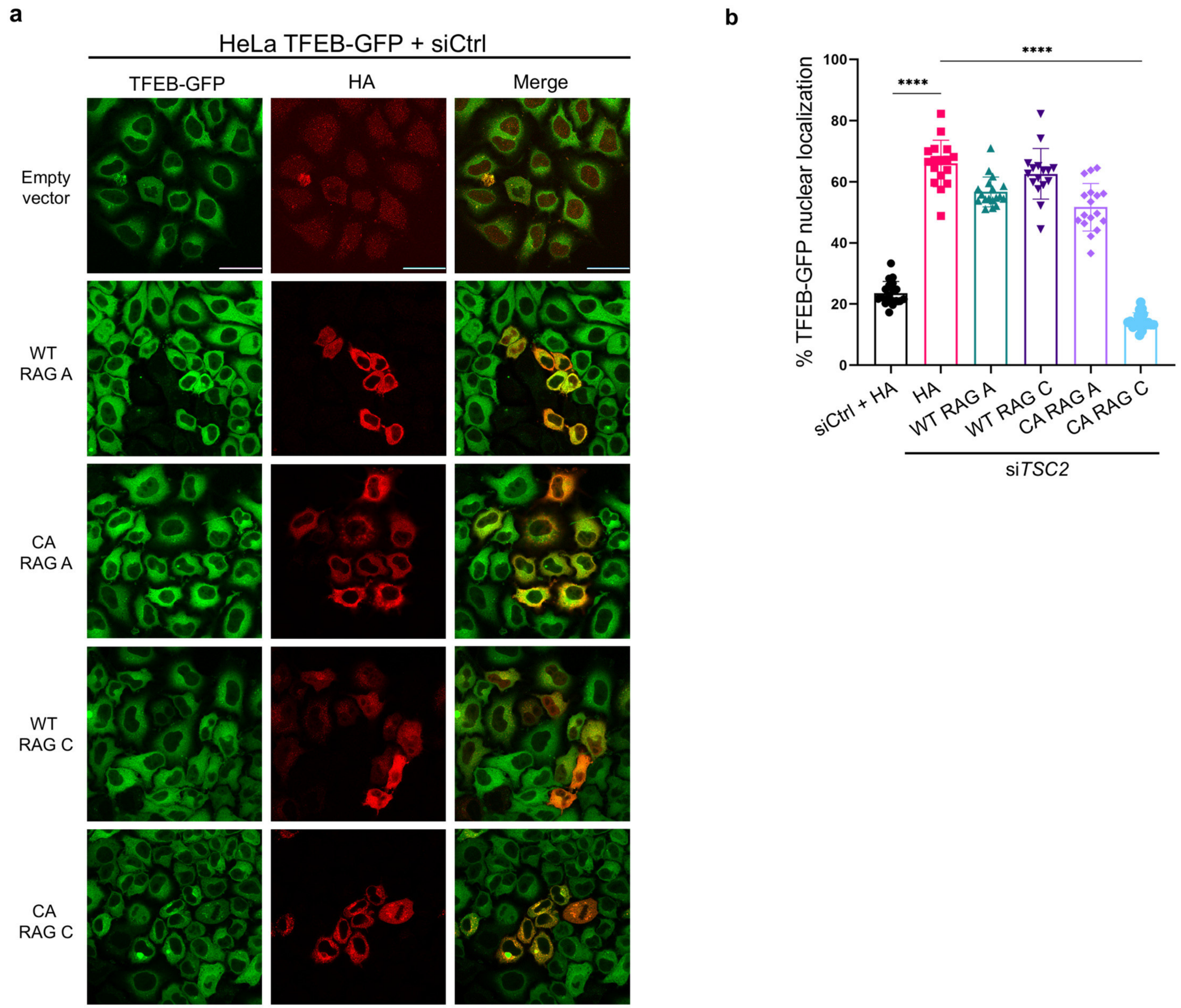

Supplementary Figure 7. CA RAGC is sufficient to induce cytoplasmic TFEB-GFP in cells with *TSC2* downregulation

a, HeLa-TFEB-GFP cells transfected with *Ctrl* siRNA for 72 hours and WT RAGA, CA RAG A, WT RAGC, or CA RAGC for 48 hr, performed in parallel to the experiment in Figure 4b (n=3 biological replicates per condition). **b,** Percentage of nuclear/cytoplasmic GFP-TFEB in cells from Figure 4b as quantified using ImageJ (n=3 random fields per condition, 15-20 HA-positive cells were analyzed in all experimental groups). Scale bars= 50 μm.

Graphs are presented as mean ± SD. Statistical analyses were performed using one-way ANOVA, ****$p<0.0001$.

Source data are provided as a Source data file.

Supplementary Figure 8

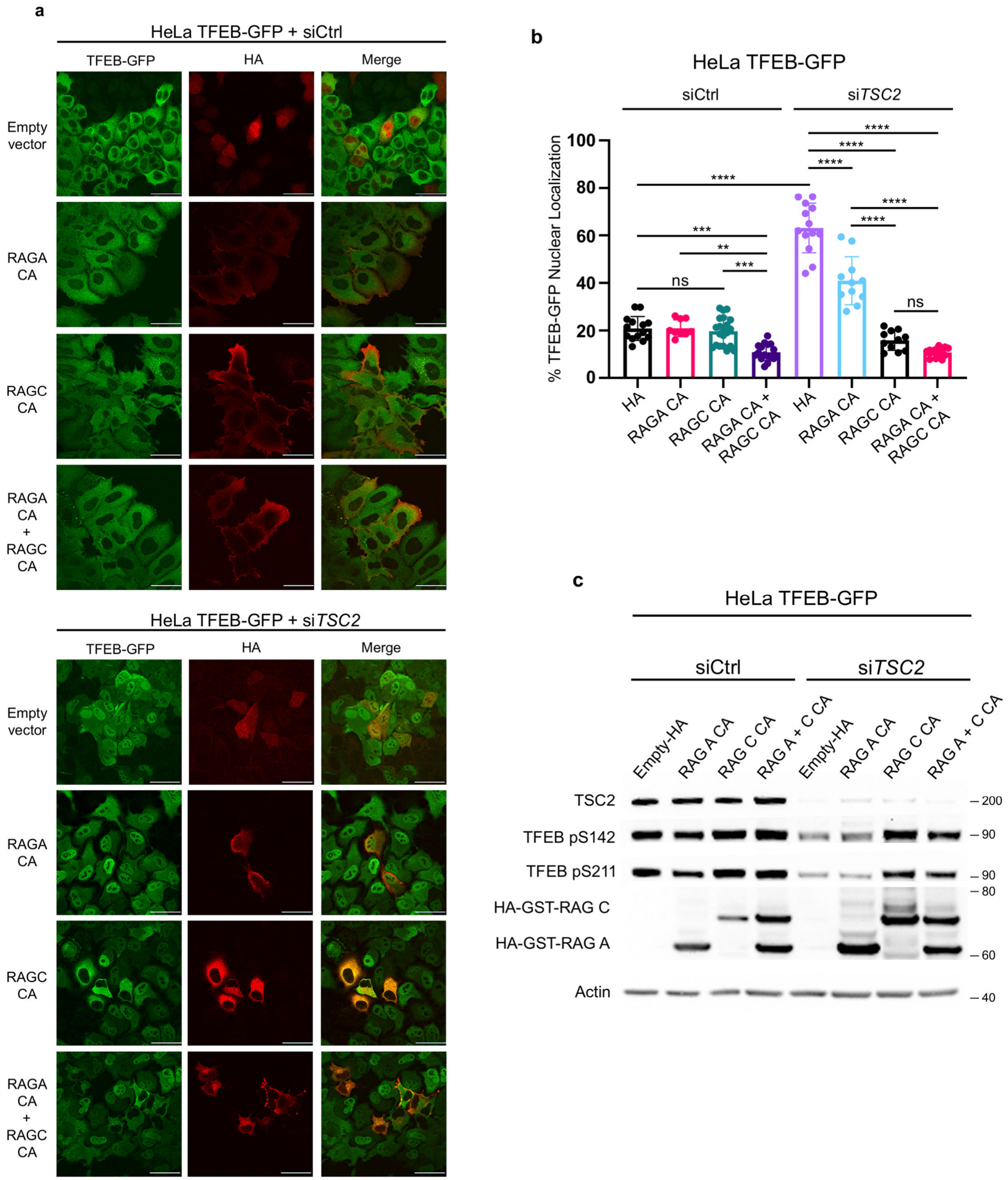

Supplementary Figure 8. CA RagC alone is as efficient as CA RAGA/CA RAGC combination to induce cytoplasmic TFEB-GFP in cells with *TSC2* downregulation

a, b, Immunofluorescent analysis of HeLa-TFEB-GFP cells treated with *Ctrl* siRNA or *TSC2* siRNA for 72 hr, transfected with CA RAGA, CA RAGC or both for 48 hr **(a)**. Percentage of nuclear/cytoplasmic TFEB-GFP in HA-positive cells, quantified using ImageJ (n=3 random images per condition, 10-15 HA-positive cells were analyzed in all experimental groups) **(b)**.**c,** Immunoblot analysis of cells treated as in **a** with indicated antibodies. Scale bars= 50 µm. Graphs are presented as mean ± SD. Statistical analyses were performed using one-way ANOVA, ** $p<0.01$, ***$p<0.001$, ****$p<0.0001$, ns= non-significant. Source data are provided as a Source data file.

Supplementary Figure 9

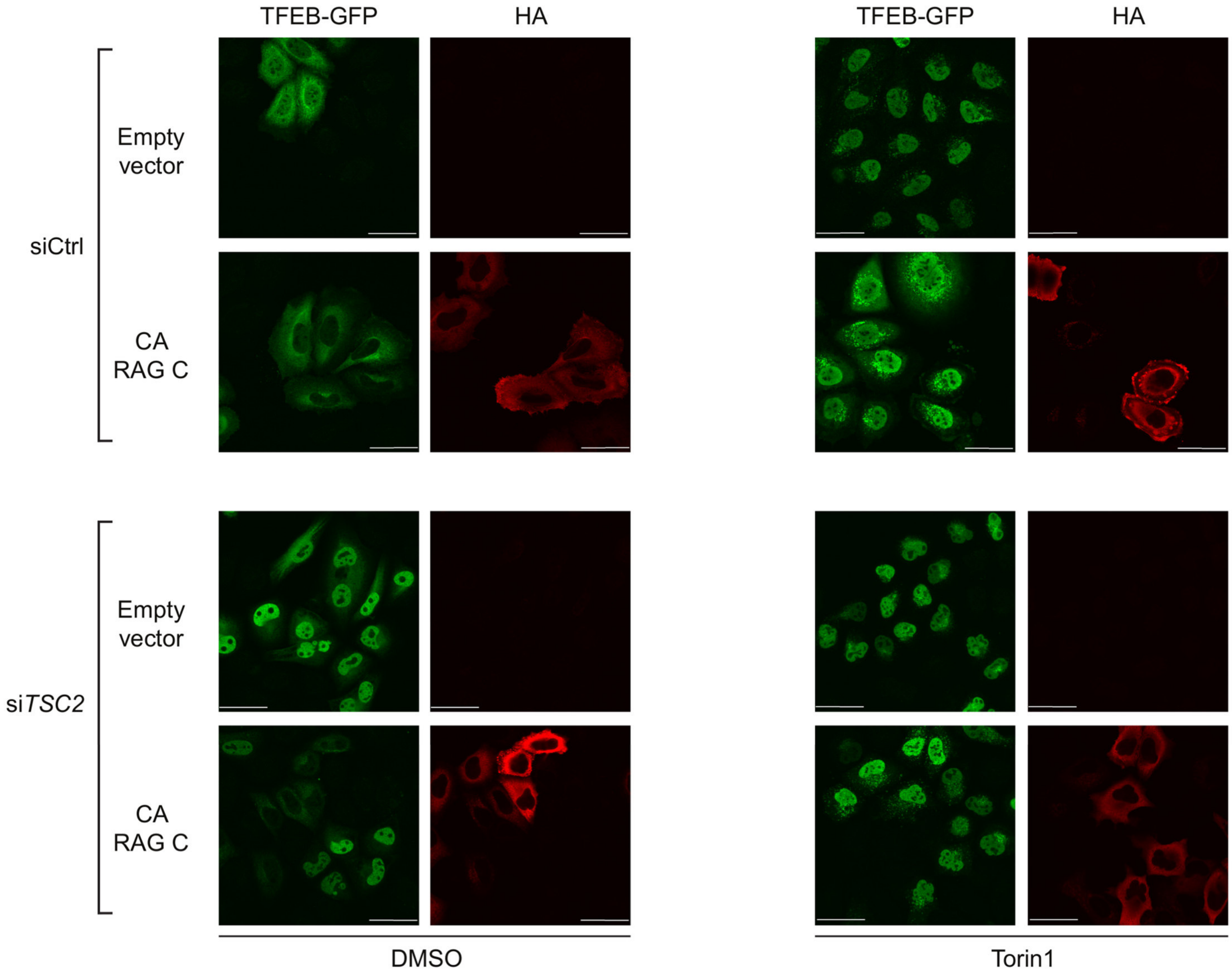

Supplementary Figure 9. Torin1 treatment localizes TFEB to the nucleus in *TSC2*-deficient cells expressing constitutively active RAGC

Immunofluorescent analysis of HeLa-TFEB-GFP cells after siRNA downregulation for 72hr transfected with constitutively active RAGC (RAGC S75N) for 48 hours and treated with Dmso or Torin1 (250nM) for six hours (n=3 biological replicates per condition). Scale bars= 50 µm.

Figure 1i

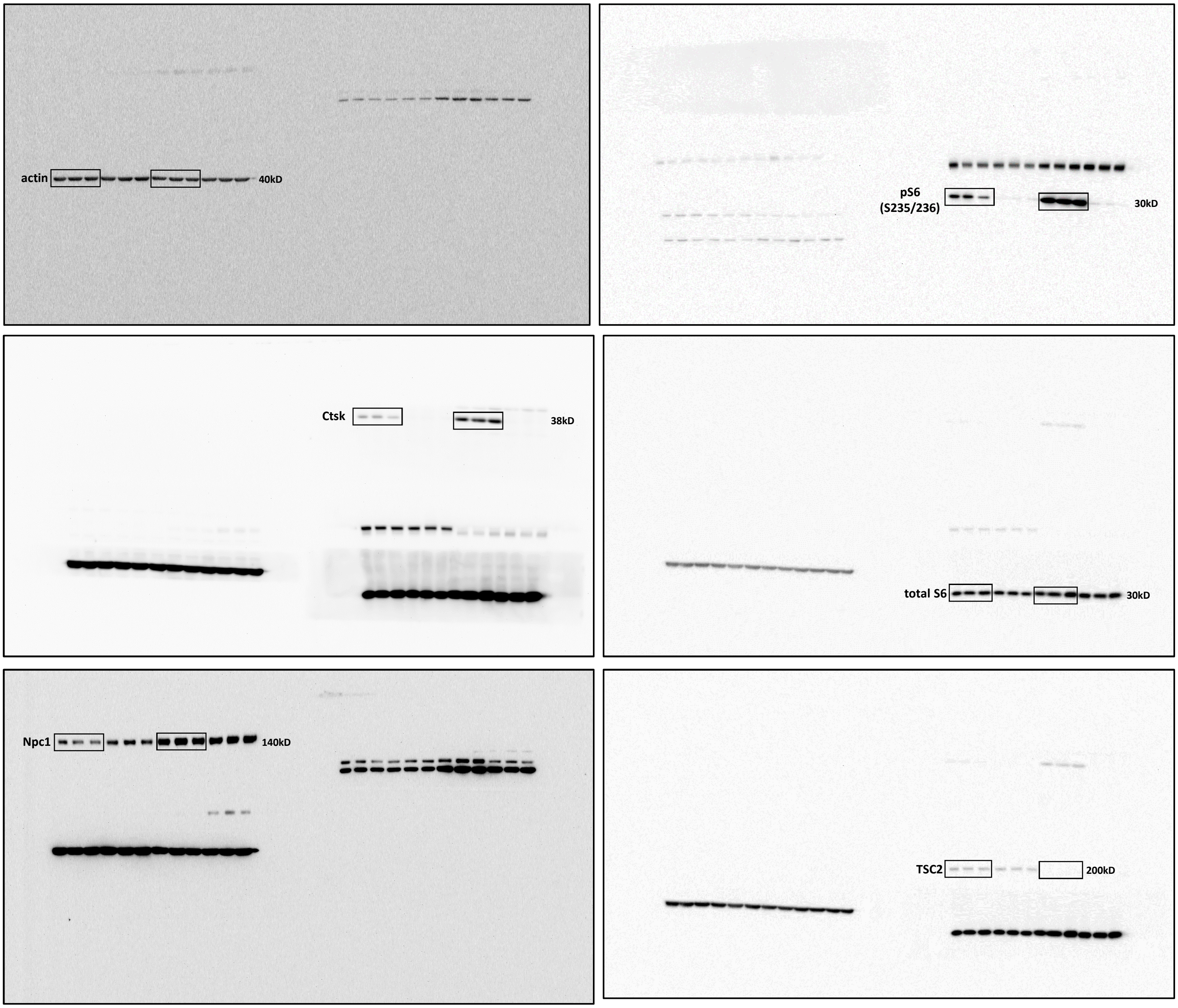
actin
40kD
pS6
(S235/236)
30kD
Ctsk
38kD
total S6
30kD
Npc1
140kD
TSC2
200kD

Figure 2f

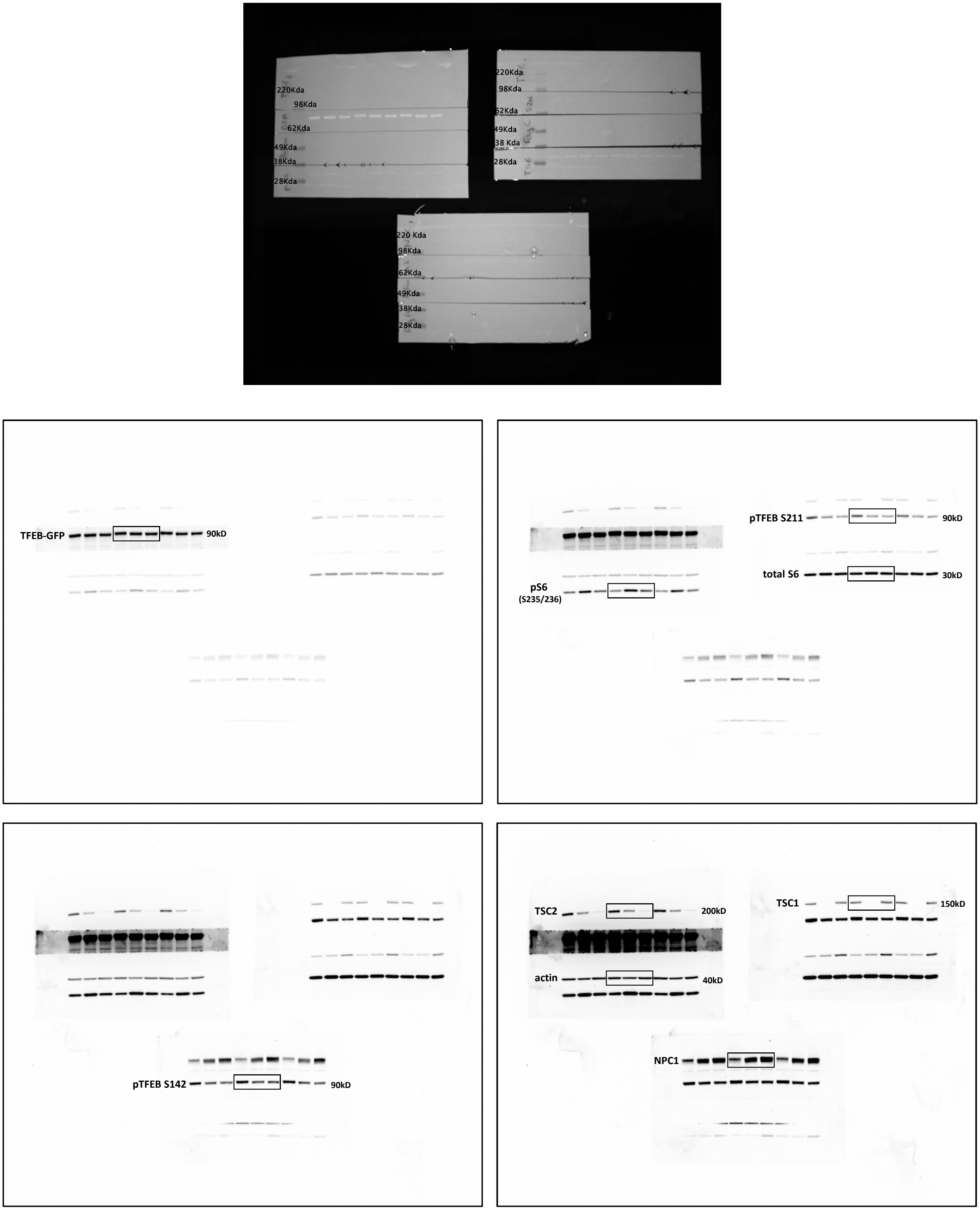
220Kda
98Kda
62Kda
49Kda
38Kda
28Kda
220Kda
98Kda
62Kda
49Kda
38 Kda
28Kda
220 Kda
98Kda
62Kda
49Kda
38Kda
28Kda
TFEB-GFP
90kD
pTFEB S211
90kD
total S6
30kD
pS6
(S235/236)
pTFEB S142
90kD
TSC2
200kD
TSC1
150kD
actin
40kD
NPC1

Figure 3b

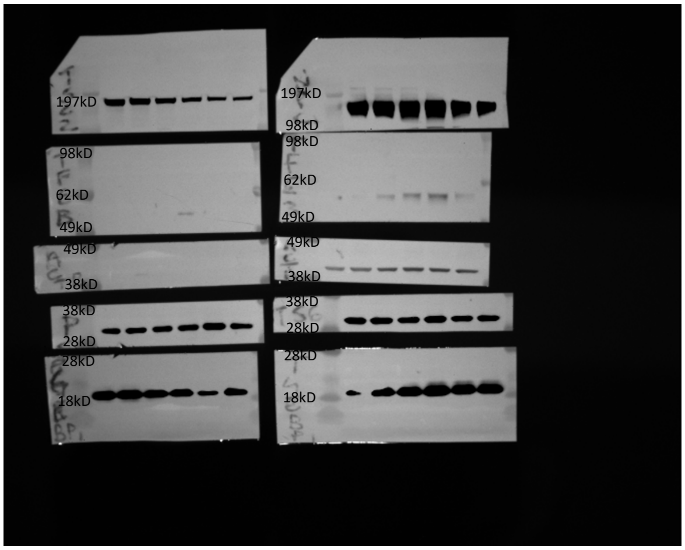
197kD
98kD
62kD
49kD
49kD
38kD
38kD
28kD
28kD
18kD
197kD
98kD
98kD
62kD
49kD
49kD
38kD
38kD
28kD
28kD
18kD

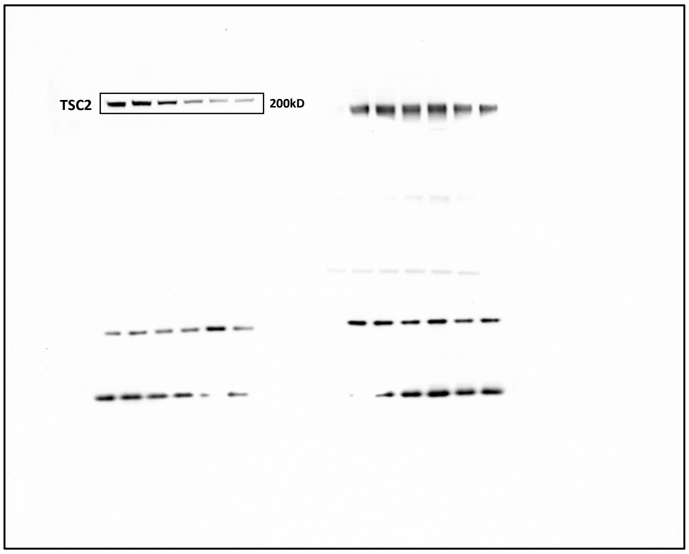
TSC2
200kD

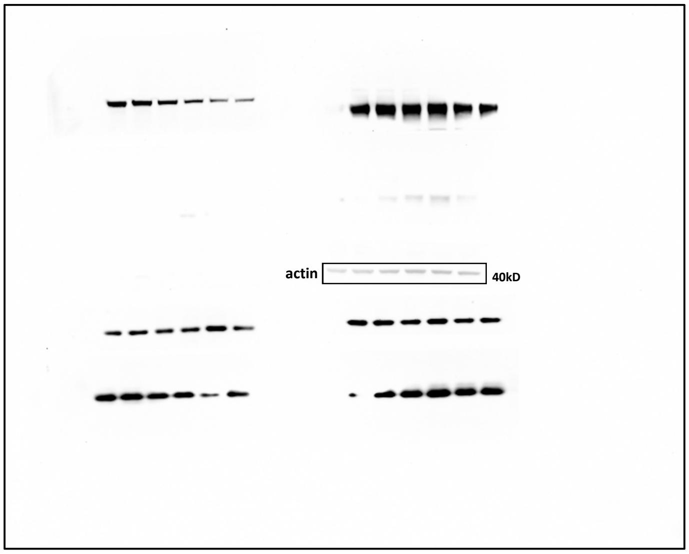
actin
40kD

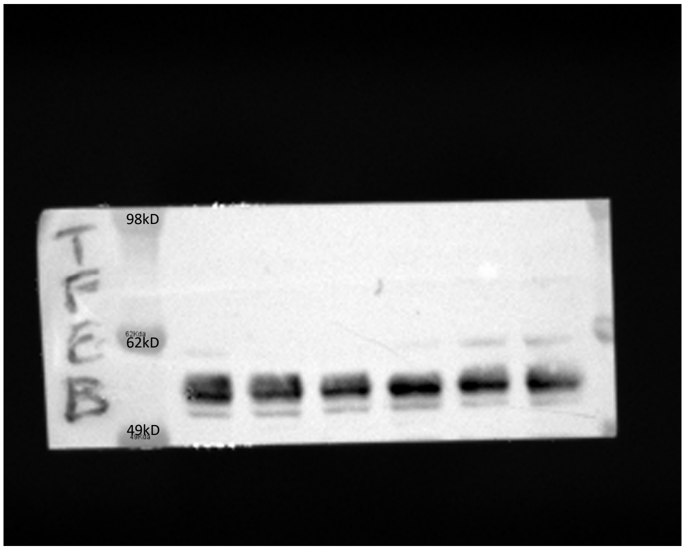
98kD
62kD
49kD

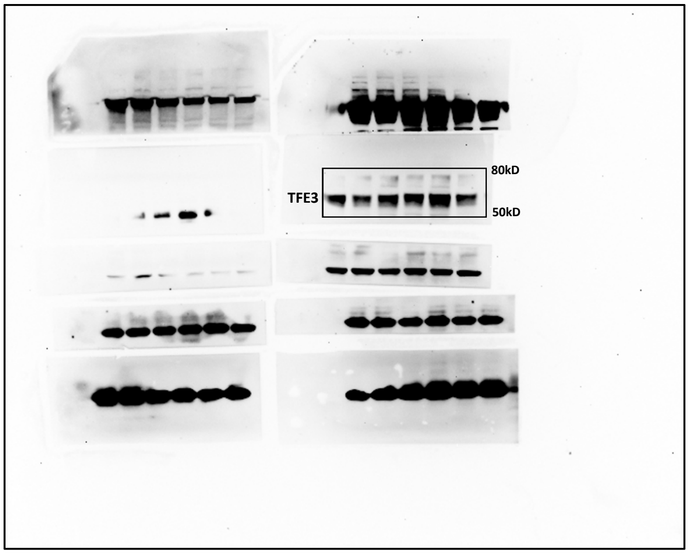
80kD
TFE3
50kD

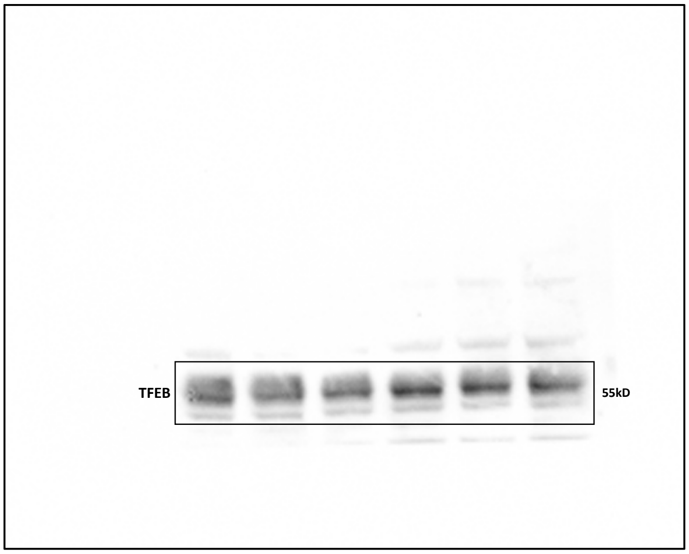
TFEB
55kD

Figure 3c

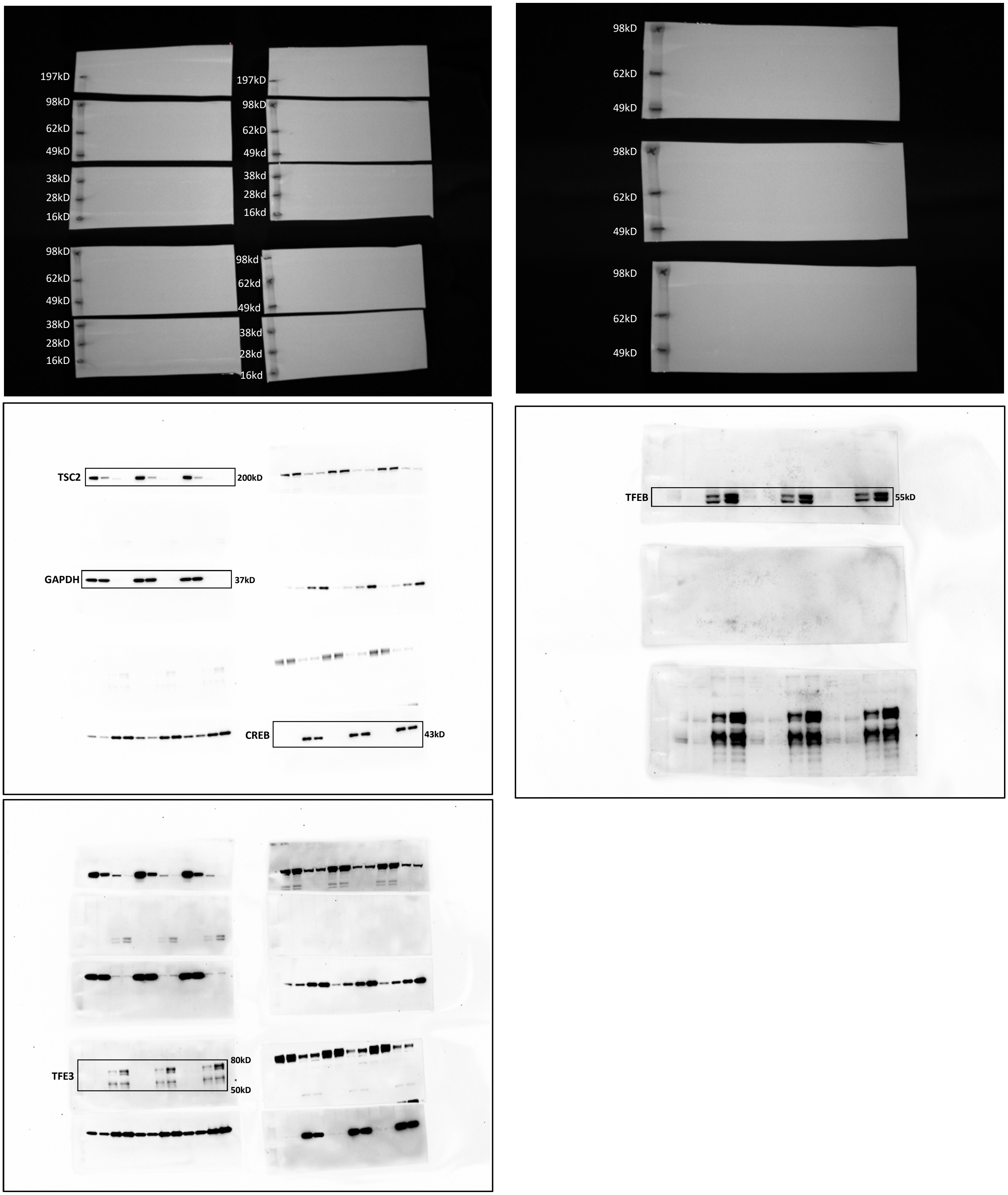

197kD
98kD
62kD
49kD
38kD
28kD
16kD
197kD
98kD
62kD
49kd
38kd
28kd
16kd
98kD
62kD
49kD
38kD
28kD
16kD
98kd
62kd
49kd
38kd
28kd
16kd
98kD
62kD
49kD
98kD
62kD
49kD
98kD
62kD
49kD
TSC2
200kD
GAPDH
37kD
CREB
43kD
TFEB
55kD
TFE3
80kD
50kD

Figure 3e

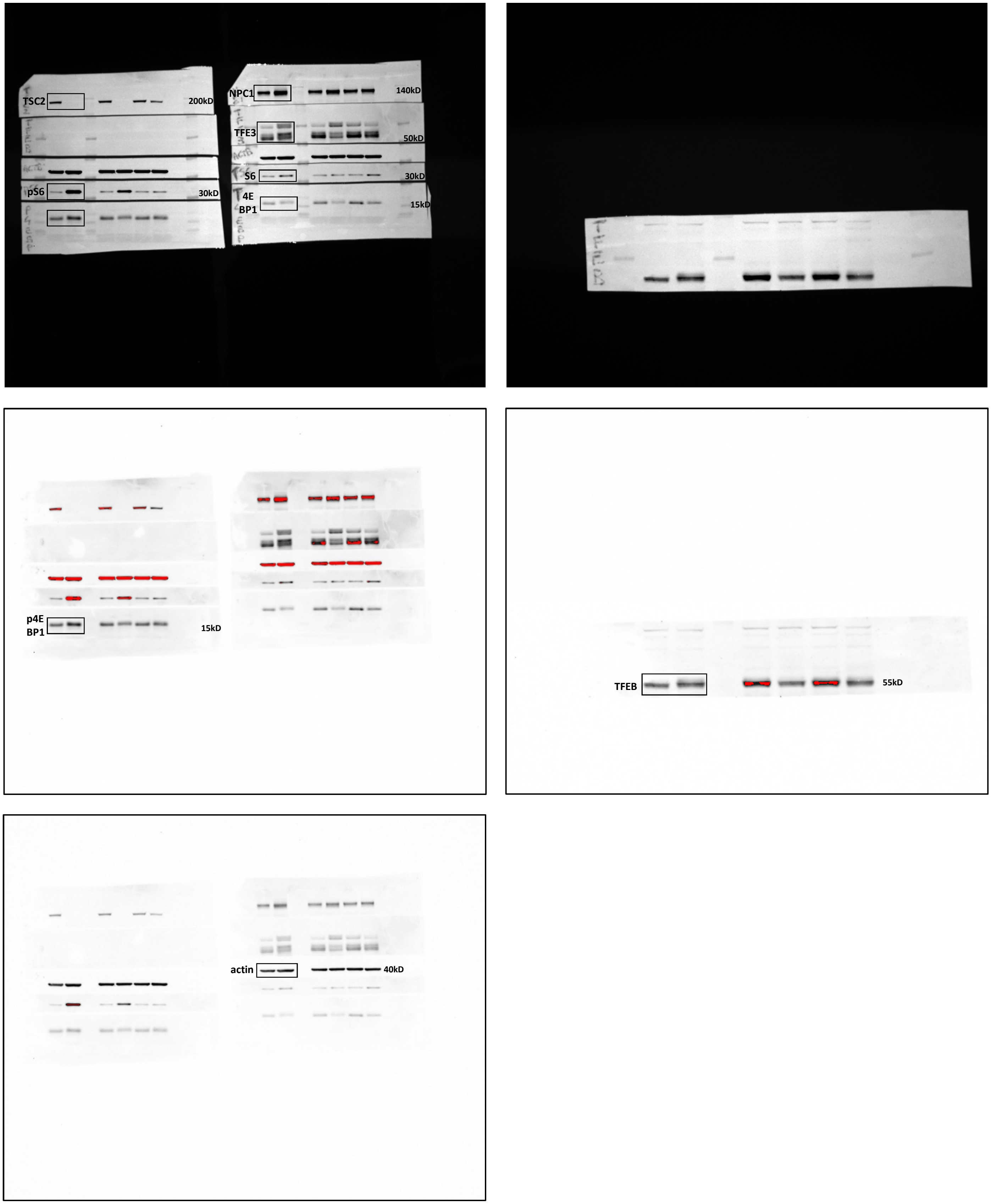
TSC2
200kD
NPC1
140kD
TFE3
50kD
pS6
30kD
S6
30kD
4E
BP1
15kD
p4E
BP1
15kD
TFEB
55kD
actin
40kD

Figure 3f

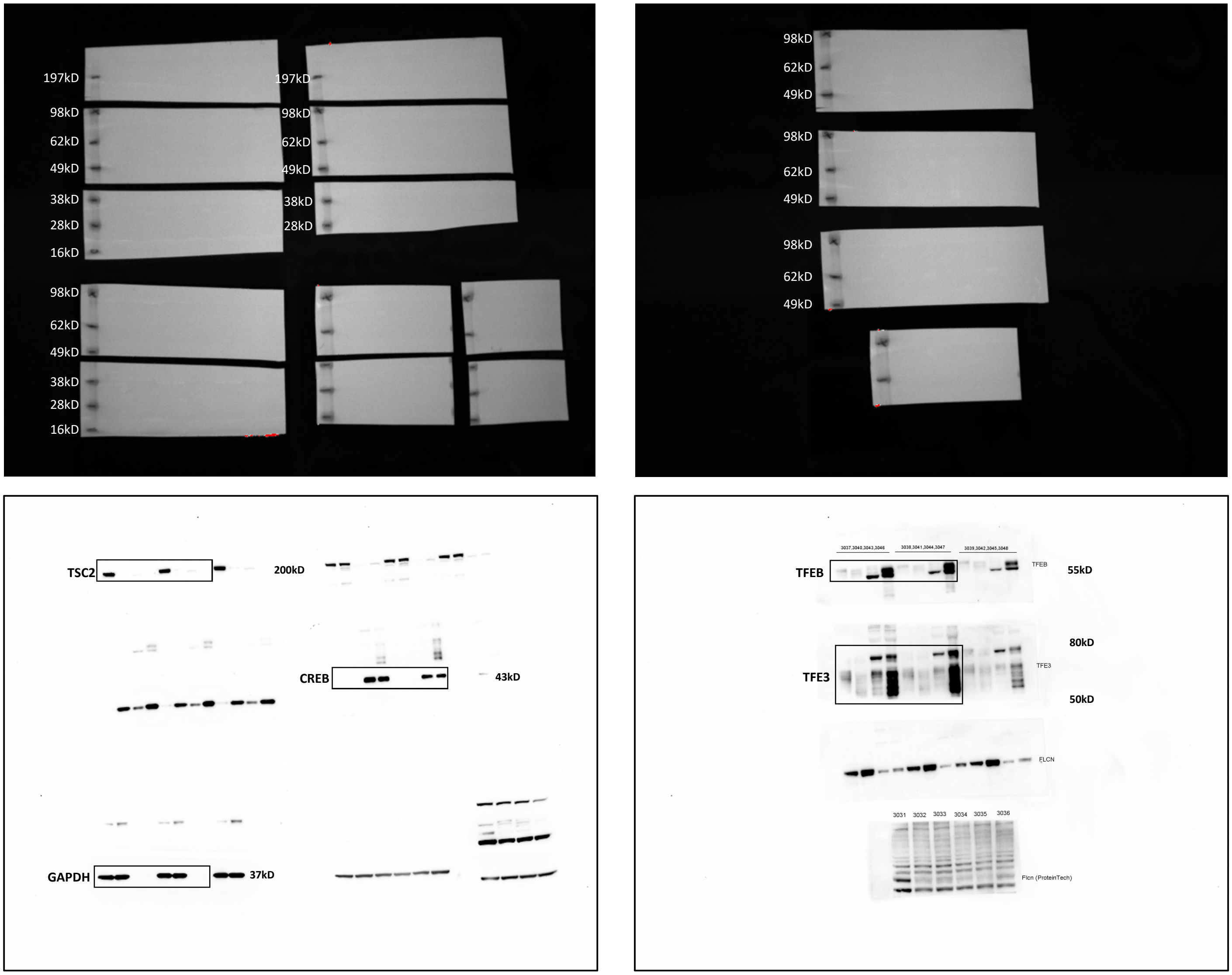

197kD
98kD
62kD
49kD
38kD
28kD
16kD
197kD
98kD
62kD
49kD
38kD
28kD
98kD
62kD
49kD
38kD
28kD
16kD
98kD
62kD
49kD
98kD
62kD
49kD
98kD
62kD
49kD
TSC2
200kD
CREB
43kD
GAPDH
37kD
3037,3040,3043,3046
3038,3041,3044,3047
3039,3042,3045,3048
TFEB
TFEB
55kD
80kD
TFE3
TFE3
50kD
FLCN
3031
3032
3033
3034
3035
3036
Flcn (ProteinTech)

Figure 4a

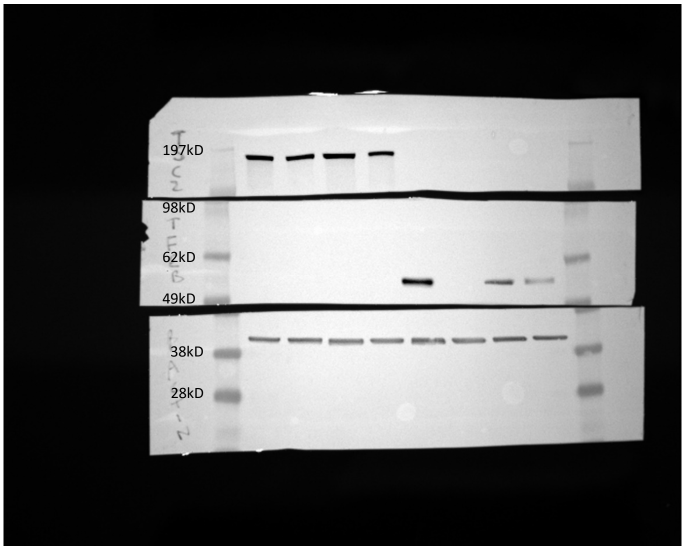
197kD
98kD
62kD
49kD
38kD
28kD

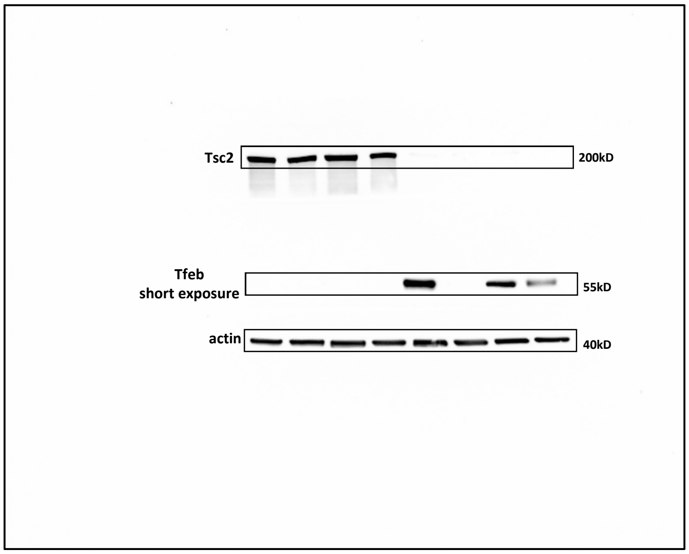
Tsc2
200kD
Tfeb
short exposure
55kD
actin
40kD

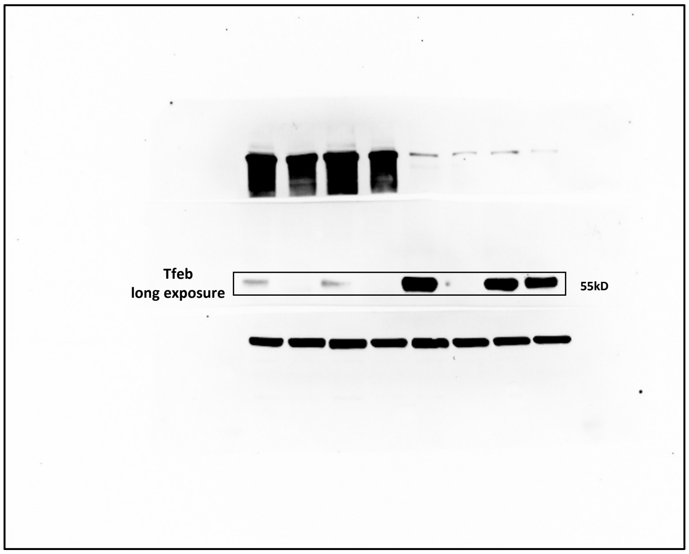
Tfeb
long exposure
55kD

Figure 5e

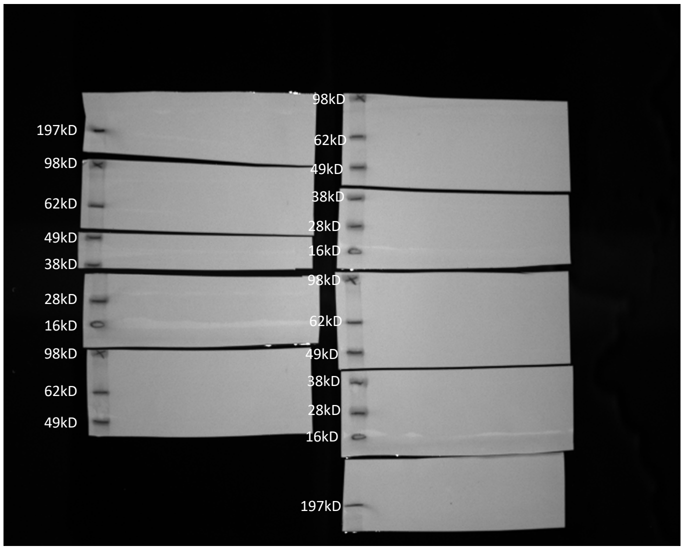
197kD
98kD
62kD
49kD
38kD
28kD
16kD
98kD
62kD
49kD
98kD
62kD
49kD
38kD
28kD
16kD
98kD
62kD
49kD
38kD
28kD
16kD
197kD

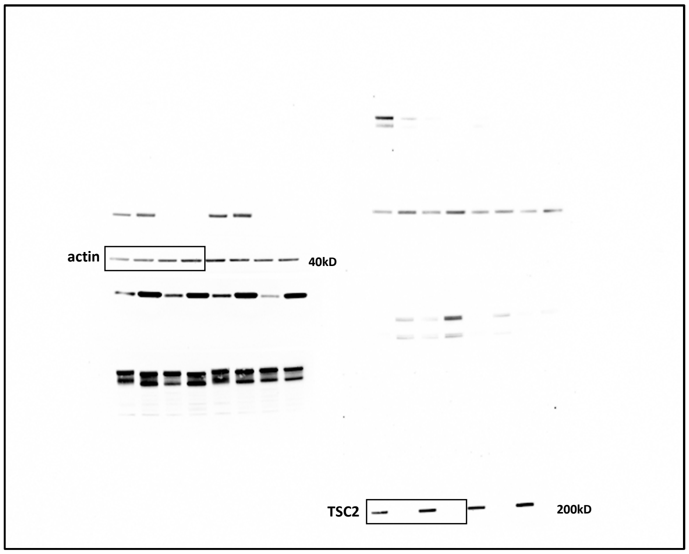
actin
40kD
TSC2
200kD

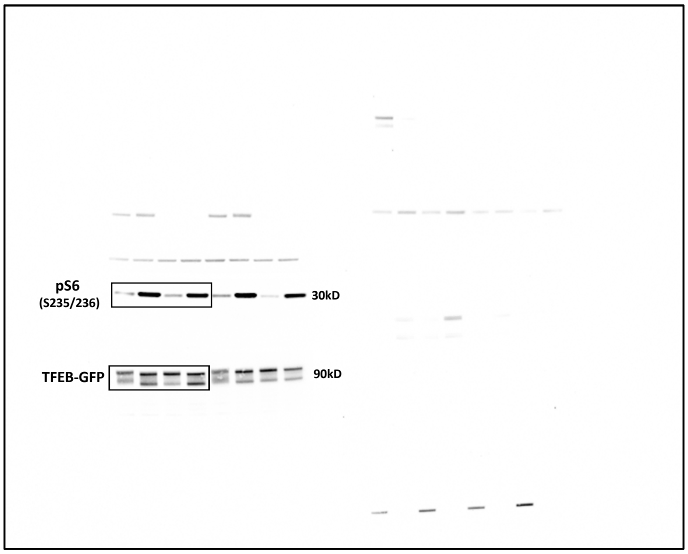
pS6
(S235/236)
30kD
TFEB-GFP
90kD

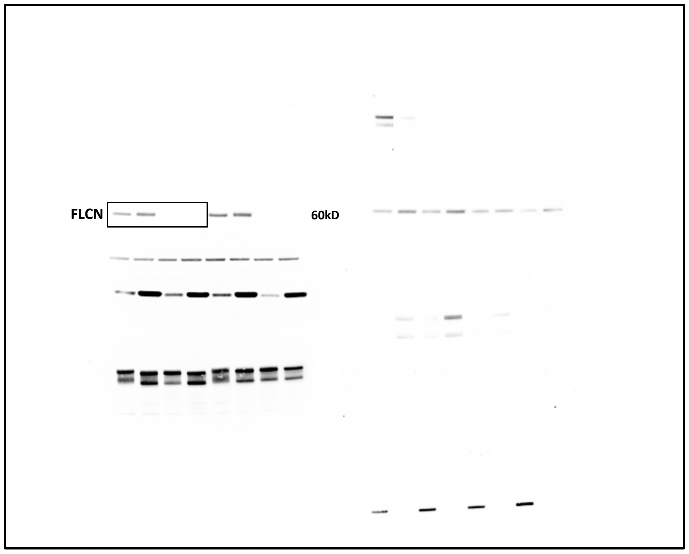
FLCN
60kD

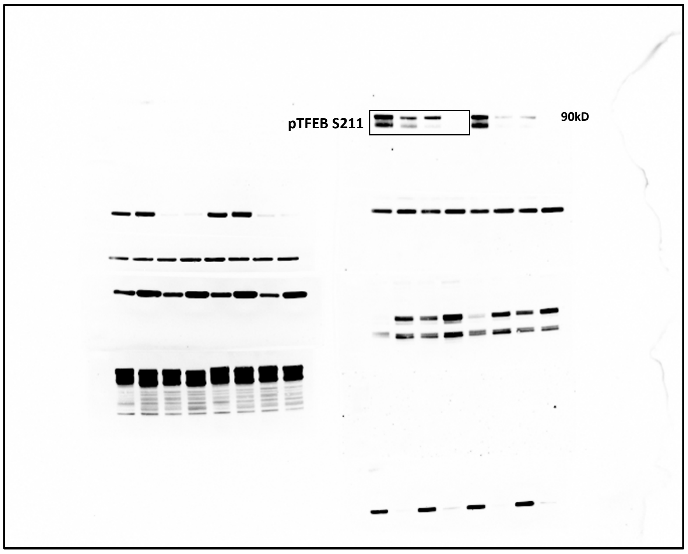
pTFEB S211
90kD

Figure 5h

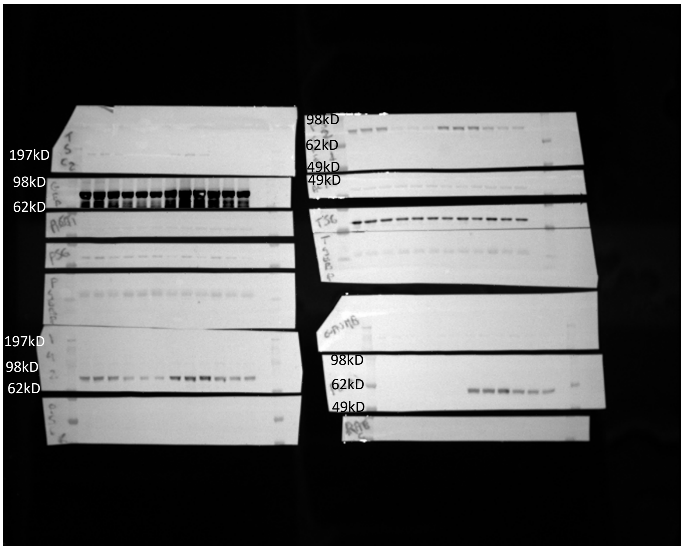

197kD
98kD
62kD
197kD
98kD
62kD
98kD
62kD
49kD
49kD
98kD
62kD
49kD

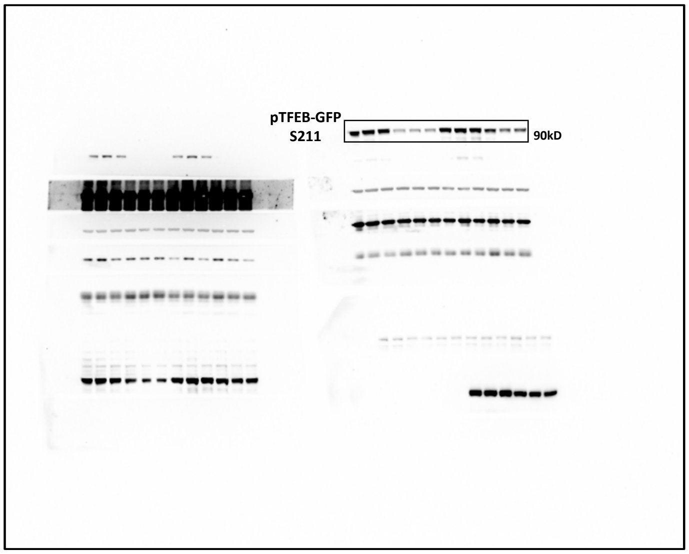

pTFEB-GFP
S211
90kD

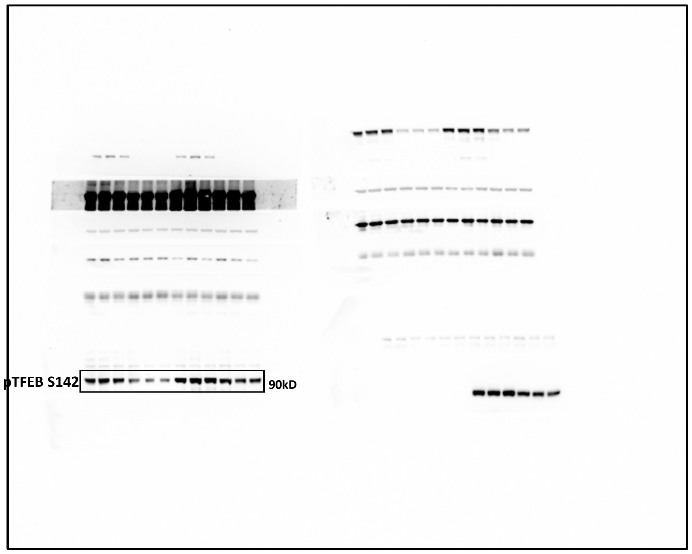

pTFEB S142
90kD

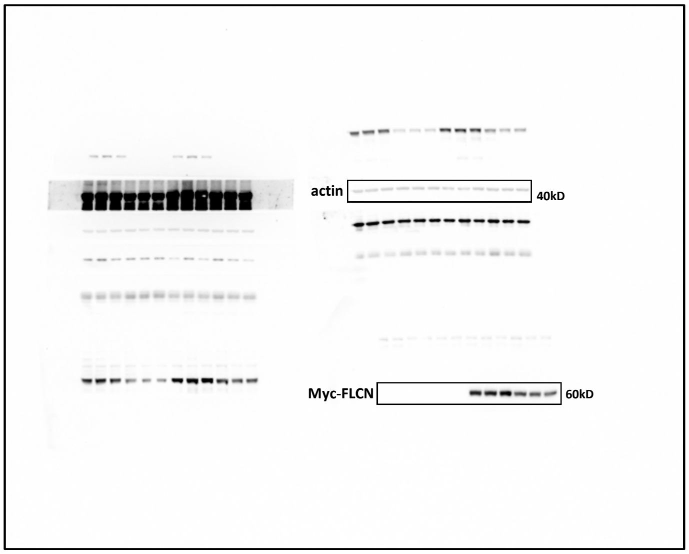

actin
40kD
Myc-FLCN
60kD

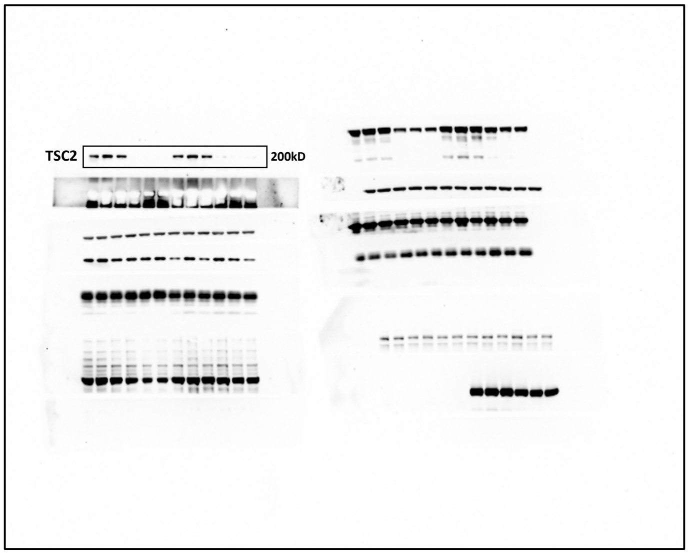

TSC2
200kD

Figure 5h

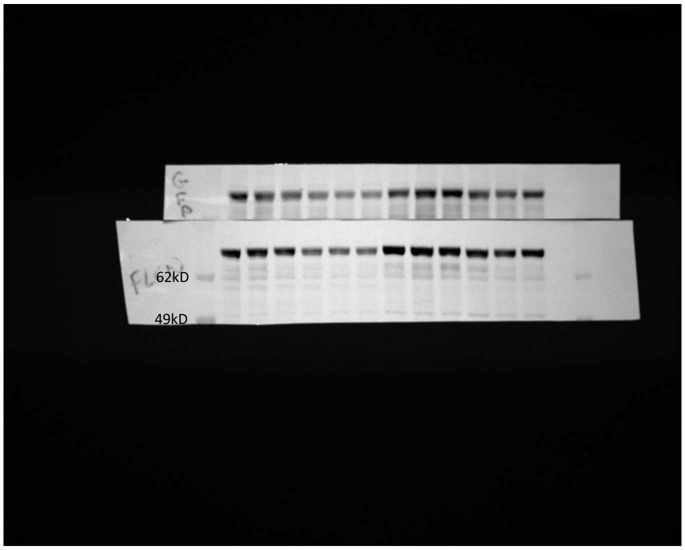

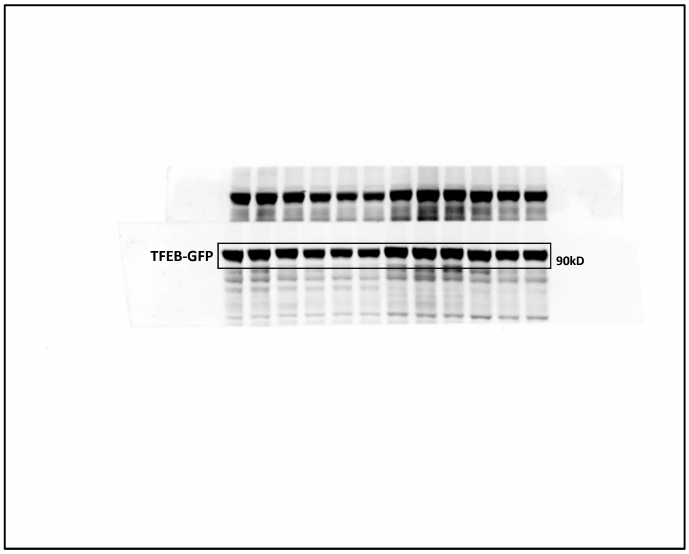

Figure 6c

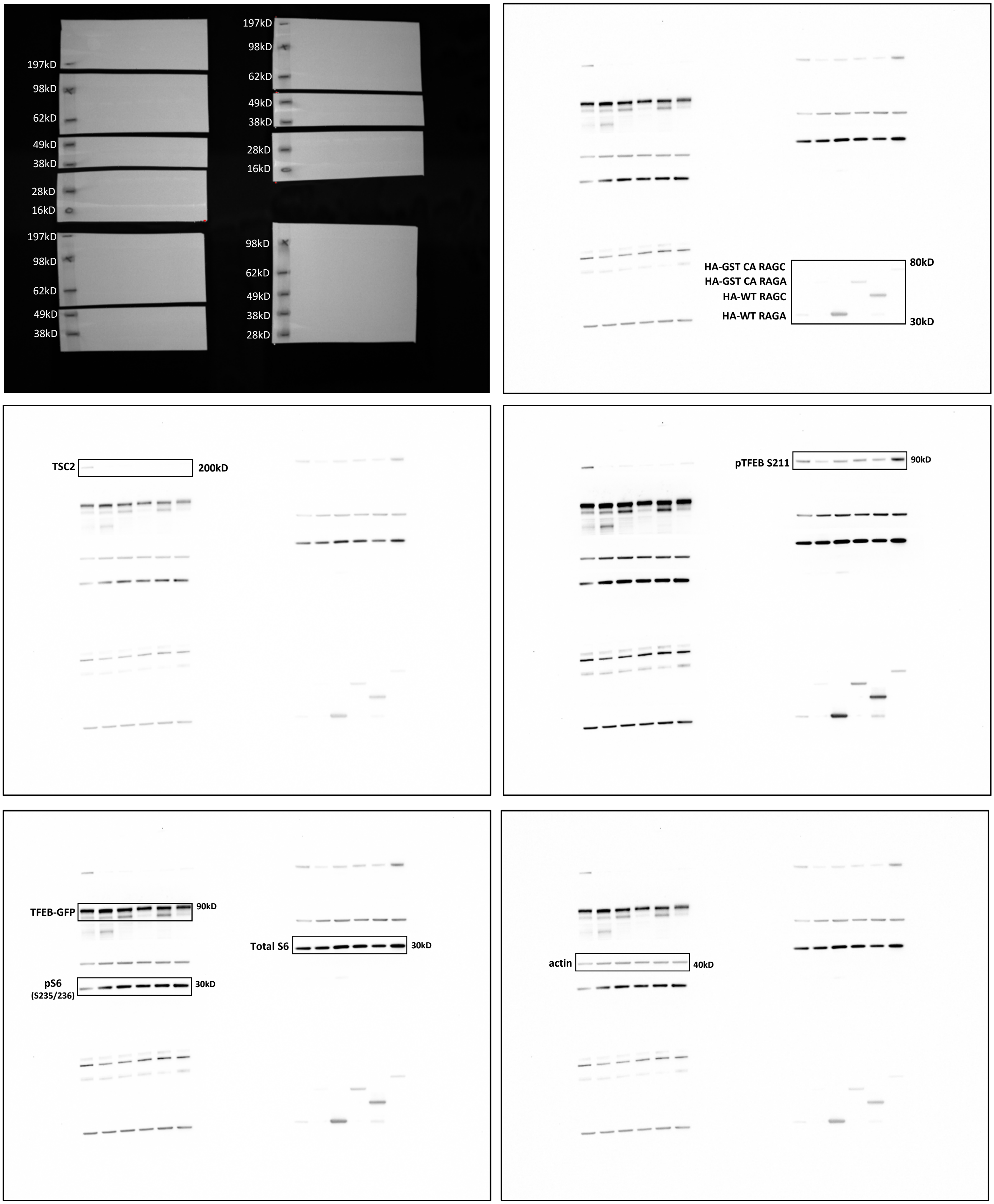
197kD
98kD
62kD
49kD
38kD
28kD
16kD
HA-GST CA RAGC
HA-GST CA RAGA
HA-WT RAGC
HA-WT RAGA
80kD
30kD
TSC2
200kD
pTFEB S211
90kD
TFEB-GFP
90kD
Total S6
30kD
pS6
(S235/236)
30kD
actin
40kD

Supplementary Figure 4b

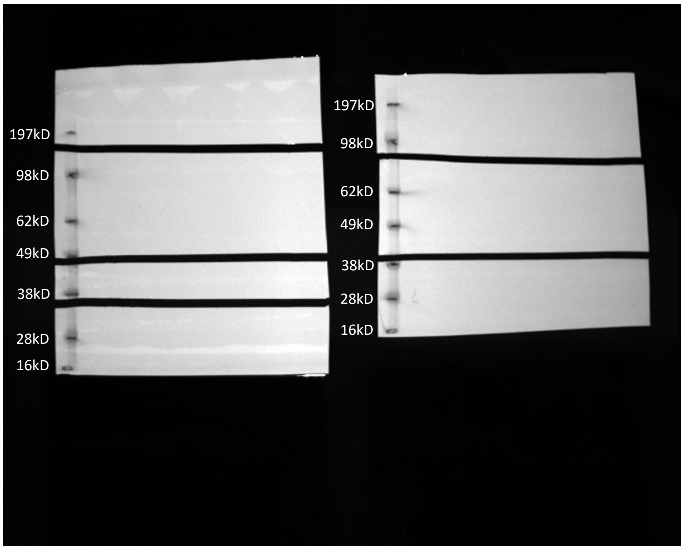

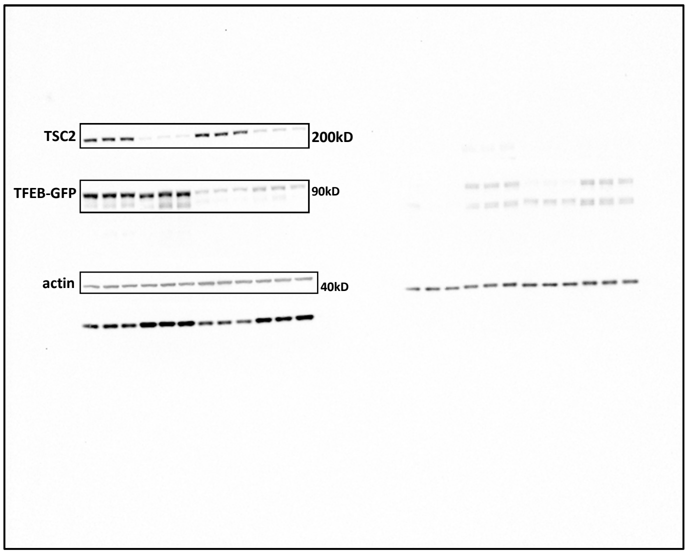

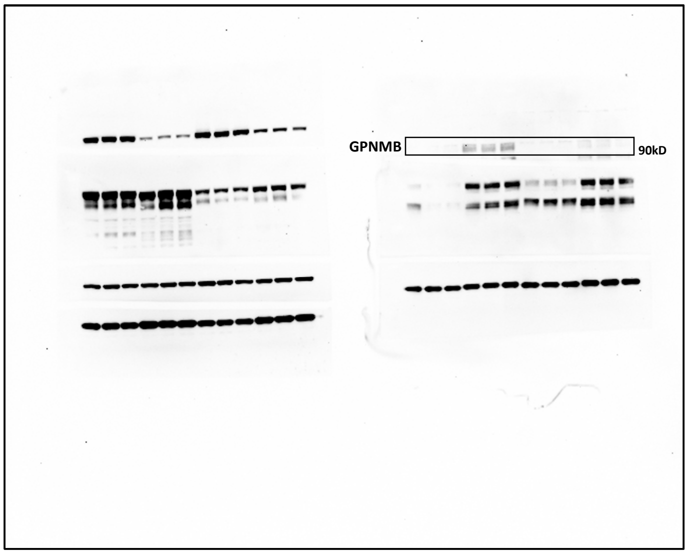

Supplementary Figure 6c

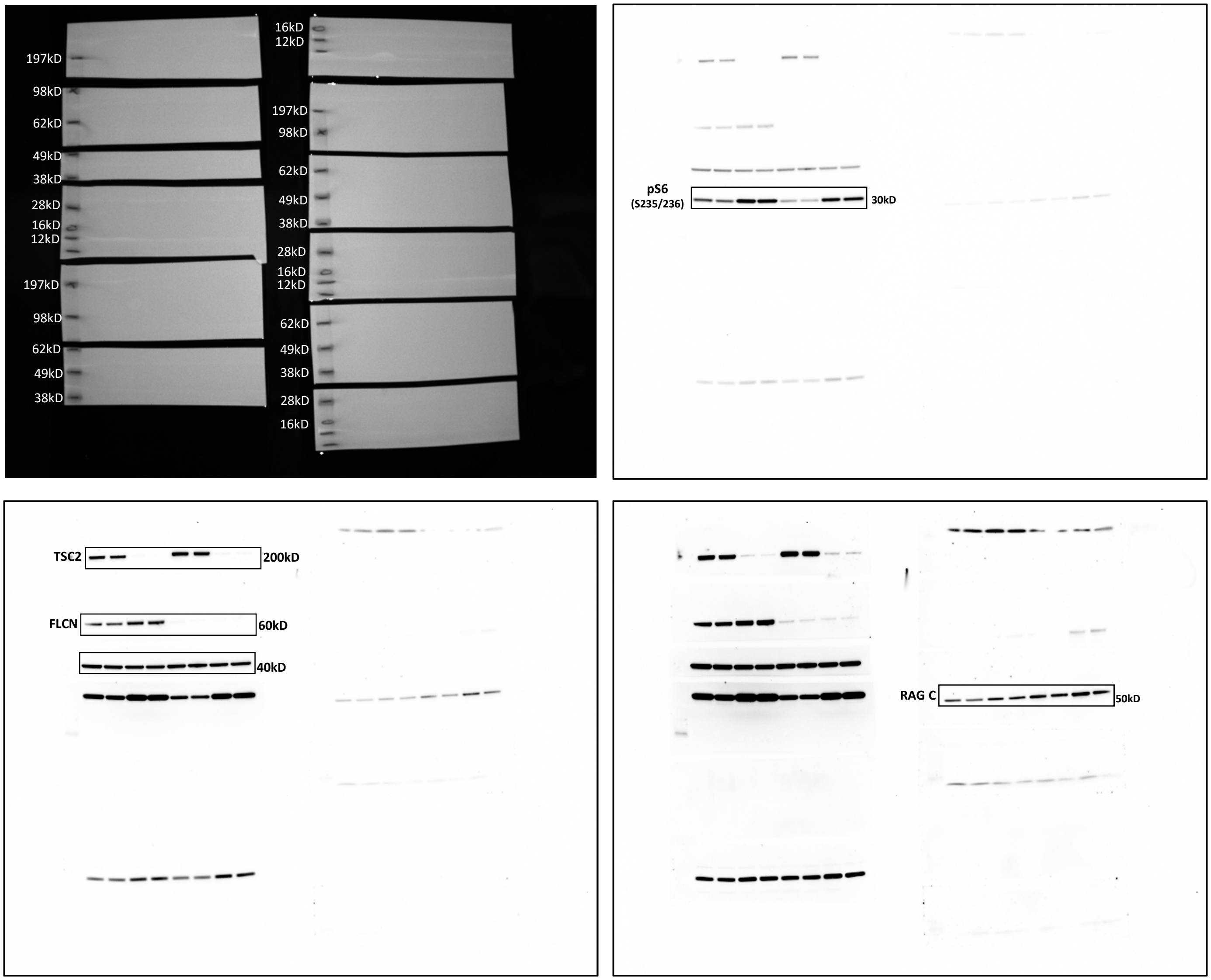

Supplementary Figure 8c

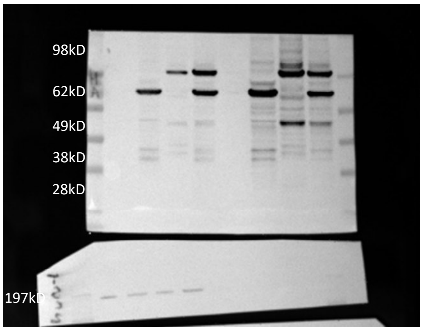

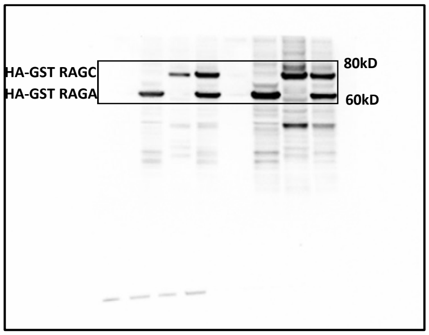

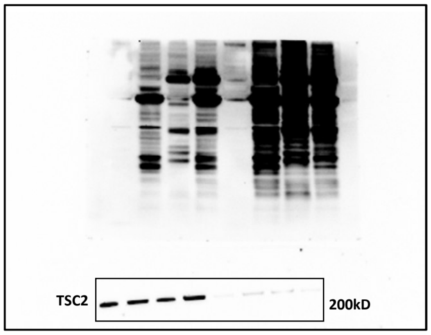

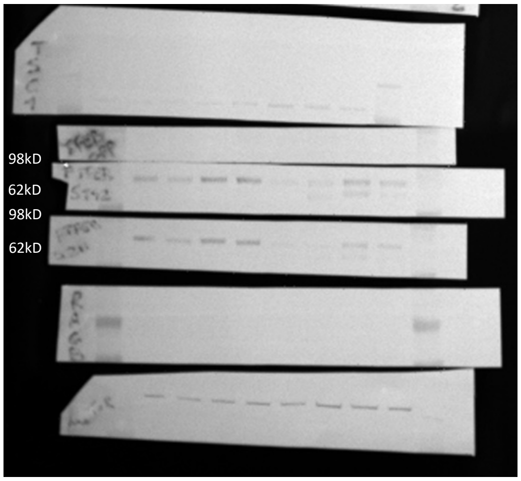

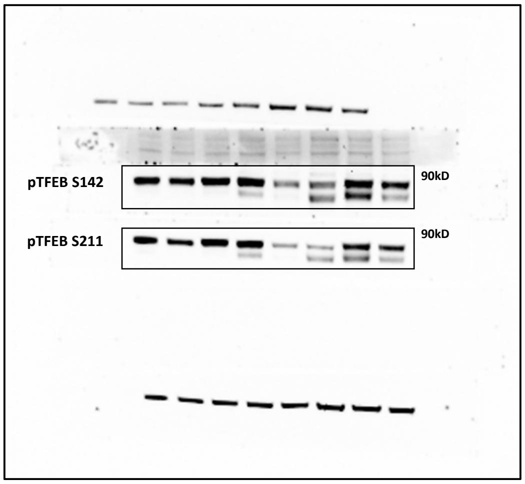

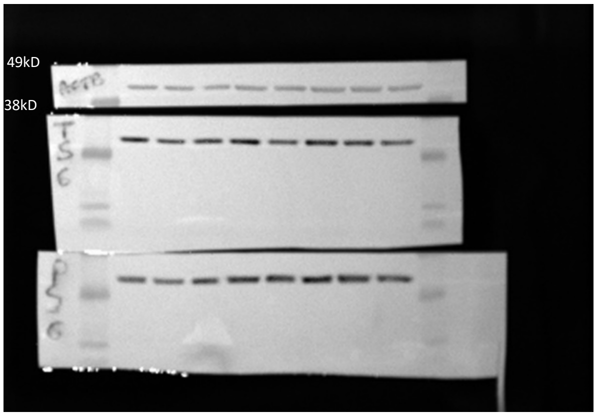

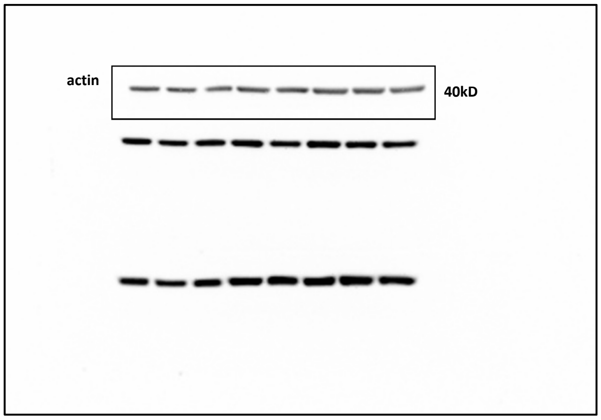

Supplementary Table 1. List of RT-PCR gene expression assays used in the study (all from ThermoFisher).

Npc1	Mm00435300_m1
Npc2	Mm00499230_m1
Hexa	Mm00599877_m1
Ctsk	Mm00484039_m1
TSC2	Hs01020387_m1
GPNMB	Hs01095679_m1
TFEB	Hs00292981_m1
CTSK	Hs00166156_m1
CTSA	Hs00264902_m1
CTSB	Hs00947439_m1
MCOLN1	Hs01100653_m1
NPC1	Hs00264835_m1
FLCN	Hs00376065_m1
RRAGC	Hs00900846_m1
RRAGD	Hs00222001_m1
ATP6V0D2	Hs00403032_m1
IL33	Hs01125943_m1
Actb	4352341E
ACTB	4326315E
