# Supplementary material for: TSC2 regulates lysosome biogenesis via a non-canonical RAGC and TFEB-dependent mechanism
Source: Nat Commun. 2021 Jul 12;12:4245. doi: 10.1038/s41467-021-24499-6 (PMC8275687; doi:10.1038/s41467-021-24499-6)
Supplement: Supplementary file 3 — Reporting Summary [file 41467_2021_24499_MOESM3_ESM.pdf]

nature research

Corresponding author(s): Elizabeth P. Henske

Last updated by author(s): 05/21/2021

Reporting Summary

Nature Research wishes to improve the reproducibility of the work that we publish. This form provides structure for consistency and transparency in reporting. For further information on Nature Research policies, see our Editorial Policies and the Editorial Policy Checklist.

Statistics

For all statistical analyses, confirm that the following items are present in the figure legend, table legend, main text, or Methods section.

n/a	Confirmed	
☐	☒	The exact sample size (*n*) for each experimental group/condition, given as a discrete number and unit of measurement
☐	☒	A statement on whether measurements were taken from distinct samples or whether the same sample was measured repeatedly
☐	☒	The statistical test(s) used AND whether they are one- or two-sided<br>*Only common tests should be described solely by name; describe more complex techniques in the Methods section.*
☒	☐	A description of all covariates tested
☐	☒	A description of any assumptions or corrections, such as tests of normality and adjustment for multiple comparisons
☐	☒	A full description of the statistical parameters including central tendency (e.g. means) or other basic estimates (e.g. regression coefficient) AND variation (e.g. standard deviation) or associated estimates of uncertainty (e.g. confidence intervals)
☐	☒	For null hypothesis testing, the test statistic (e.g. F, t, r) with confidence intervals, effect sizes, degrees of freedom and P value noted<br>*Give P values as exact values whenever suitable.*
☒	☐	For Bayesian analysis, information on the choice of priors and Markov chain Monte Carlo settings
☒	☐	For hierarchical and complex designs, identification of the appropriate level for tests and full reporting of outcomes
☒	☐	Estimates of effect sizes (e.g. Cohen's d, Pearson's r), indicating how they were calculated

Our web collection on statistics for biologists contains articles on many of the points above.

Software and code

Policy information about availability of computer code

Data collection	Electron microscopy images were captured using CM10 transmission electron microscope. Confocal images were captured using Olympus FV10-FSW software package (v 4.1). IHC images were captured using Keyence BZ-X800 microscope and its integrated viewer. Chemiluminescent images were captured using Syngene G-Box and ThermoFisher iBright 1500 imagers. GPNMB reporter activity using nano-luciferase was analyzed using Biotek Synergy HT running Gen5 v1.11.5 software. Gene expression by RT-PCR was analyzed using AppliedBiosystems Step One Plus real time PCR system (v2.3).
Data analysis	ImageJ (v 1.52p) and Cell Profiler (v 4.0.4) were used for image analysis. GraphPad Prizm (v 8.3.1) was used for statistical analysis.

For manuscripts utilizing custom algorithms or software that are central to the research but not yet described in published literature, software must be made available to editors and reviewers. We strongly encourage code deposition in a community repository (e.g. GitHub). See the Nature Research guidelines for submitting code & software for further information.

Data

Policy information about availability of data

All manuscripts must include a data availability statement. This statement should provide the following information, where applicable:

- Accession codes, unique identifiers, or web links for publicly available datasets
- A list of figures that have associated raw data
- A description of any restrictions on data availability

All data are available from the corresponding authors upon request and all unique materials generated (such as HeLa TSC2 CRISPR KO line) are readily available from the authors. Source data for Figs 1b,f,g,h,i, 2b,e,g,h,f, 3a-f, 4a-c, 5a,c-i, 6c and Suppl Figs 1b,e, 4a-c, 5a,b, 6a-c, 7b, 8b,c are provided with this paper in a Source data file and a separate supplementary pdf file with uncropped western blots.

Field-specific reporting

Please select the one below that is the best fit for your research. If you are not sure, read the appropriate sections before making your selection.

☒ Life sciences ☐ Behavioural & social sciences ☐ Ecological, evolutionary & environmental sciences

For a reference copy of the document with all sections, see nature.com/documents/nr-reporting-summary-flat.pdf

Life sciences study design

All studies must disclose on these points even when the disclosure is negative.

Sample size	Appropriate sample sizes based on assay variability were chosen.
Data exclusions	No data were excluded from the analyses.
Replication	All experiments were repeated at least in 3 biological replicates.
Randomization	Randomization was not relevant to this study.
Blinding	The quantification of the IF and IHC experiments was conducted blindly whenever possible based on experimental condition and software-based quantification methods (ImageJ and CellProfiler) were used to prevent bias.

Reporting for specific materials, systems and methods

We require information from authors about some types of materials, experimental systems and methods used in many studies. Here, indicate whether each material, system or method listed is relevant to your study. If you are not sure if a list item applies to your research, read the appropriate section before selecting a response.

Materials & experimental systems

n/a	Involved in the study	
☐	☒	Antibodies
☐	☒	Eukaryotic cell lines
☒	☐	Palaeontology and archaeology
☐	☒	Animals and other organisms
☒	☐	Human research participants
☒	☐	Clinical data
☒	☐	Dual use research of concern

Methods

n/a	Involved in the study	
☒	☐	ChIP-seq
☒	☐	Flow cytometry
☒	☐	MRI-based neuroimaging

Antibodies

Antibodies used

Antibodies used:
Primary antibodies:
Mouse beta-actin (Millipore Sigma, A5316) 1:1000 for WB
Rabbit phosphorylated S6 ribosomal protein - pS6 Serine235/236 (Cell Signaling Technology, 2211) 1:1000 for WB
Rabbit total S6 Ribosomal protein (Cell Signaling Technology, 2217) 1:1000 for WB
Phosphorylated 4E-BP1 – p4E-BP1 Threonine 37/46 (Cell Signaling Technology, 2855) 1:1000 for WB
Rabbit total 4E-BP1 (Cell Signaling Technology, 9644) 1:1000 for WB
Rabbit TSC2 (Cell Signaling Technology, 4308) 1:1000 for WB
Rabbit TSC1 (Cell Signaling Technology, 6935) 1:1000 for WB
Rabbit TFEB (Bethyl Laboratories, A303-673A) 1:3000 for WB
Rabbit TFEB (Cell Signaling Technology, 32361) 1:1000 for WB, 1:150 for IHC, 1:200 for IF
Rabbit TFE3 (Cell Signaling Technology, 14779) 1:1000 for WB
Rabbit TFE3 (Millipore Sigma, HPA023881) 1:1000 for WB, 1:200 for IF
Rabbit Cathepsin K (Abcam, ab19027) 1:1000 for WB
Rabbit NPC1 (Abcam, ab134113) 1:1000 for WB, 1:400 for IHC
Rabbit FLCN (Cell Signaling Technology, 3697) 1:1000 for WB
Chicken GFP (Abcam, ab13970) 1:5000 for WB, 1:1000 for IF
Rabbit phosphorylated TFEB at serine 211 (S211) (Cell Signaling Technology, 37681) 1:1000 for WB
Rabbit phosphorylated TFEB at serine 142 (S142) (Millipore Sigma, ABE1971) 1:1000 for WB
Rabbit RagC (Cell Signaling Technology, 9480) 1:1000 for WB
Mouse Myc-tag (Cell Signaling Technology, 2276) 1:1000 for WB
Rabbit HA-tag (Cell Signaling Technology, 3724) 1:1000 for WB, 1:1000 for IF
Rabbit GPNMB (Cell Signaling Technology, 38313) 1:1000 for WB
Rabbit CREB (Cell Signaling Technology, 9197) 1:1000 for WB

Rabbit GAPDH (Cell Signaling Technology, 5174) 1:1000 for WB

Secondary antibodies:
ThermoFisher AlexaFluor 568 donkey anti-rabbit, #A10042, 1:1000
ThermoFisher AlexaFluor 488 donkey anti-rabbit, #A21206, 1:1000
Amersham/GE Healthcare ECL Mouse IgG, HRP-linked whole Ab (from sheep), #NA931, 1:3000
Amersham/GE Healcare ECL Rabbit IgG, HRP-linked whole Ab (from donkey), NA934, 1:3000
Abcam Goat Anti-Chicken IgY H&L (HRP), #ab97135, 1:5000

Validation

Mouse beta-actin (Millipore Sigma, A5316), 2848 citations. https://www.sigmaaldrich.com/catalog/product/sigma/a5316?lang=en&region=US&cm_sp=Insite-_-caSrpResults_srpRecs_srpModel_a5316-_-srpRecs3-1. The antibody was validated by the manufacturer for western blotting, immunohistochemistry and indirect immunofluorescence. Validation from our study: the antibody produces a band at the expected size on the western blot.
Rabbit phosphorylated S6 ribosomal protein - pS6 Serine235/236 (Cell Signaling Technology, 2211), 852 citations. https://www.cellsignal.com//products/primary-antibodies/phospho-s6-ribosomal-protein-ser235-236-antibody/2211. The antibody was validated by the manufacturer for western blotting, immunohistochemistry, immunofluorescence, immunoprecipitation and flow cytometry. Validation from our study: the antibody produces a band at the expected size on the western blot, band intensity increases with TSC2 downregulation or knock-out consistent with increased mTOR activation.
Rabbit total S6 Ribosomal protein (Cell Signaling Technology, 2217), 1143 citations. https://www.cellsignal.com//products/primary-antibodies/s6-ribosomal-protein-5g10-rabbit-mab/2217. The antibody was validated by the manufacturer for western blotting, immunohistochemistry and immunofluorescence. Validation from our study: the antibody produces a band at the expected size on the western blot.
Phosphorylated 4E-BP1 – p4E-BP1 Threonine 37/46 (Cell Signaling Technology, 2855), 1019 citations. https://www.cellsignal.com//products/primary-antibodies/phospho-4e-bp1-thr37-46-236b4-rabbit-mab/2855. The antibody was validated by the manufacturer for western blotting, immunohistochemistry, immunofluorescence and flow cytometry. Validation from our study: the antibody produces a band at the expected size on the western blot.
Rabbit total 4E-BP1 (Cell Signaling Technology, 9644), 572 citations. https://www.cellsignal.com//products/primary-antibodies/4e-bp1-53h11-rabbit-mab/9644. The antibody was validated by the manufacturer for western blotting, immunohistochemistry, immunofluorescence, immunoprecipitation and flow cytometry. Validation from our study: the antibody produces a band at the expected size on the western blot.
Rabbit TSC2 (Cell Signaling Technology, 4308), 150 citations. https://www.cellsignal.com//products/primary-antibodies/tuberin-tsc2-d93f12-xp-rabbit-mab/4308. The antibody was validated by the manufacturer for western blotting, immunofluorescence, immunoprecipitation and flow cytometry. Validation from our study: the antibody produces a band at the expected size on the western blot with a decreased intensity in human cells with siRNA downregulation (Figures 2f, 3b, 3c, 5e, 5h, 6c; Suppl Figure 4b, Suppl Fig 6c, Suppl Fig 8c,) and complete disappearance in HeLa TSC2 CRISPR KO cells (Figure 3e,f) and MEFs (Figure 4i).
Rabbit TSC1 (Cell Signaling Technology, 6935), 44 citations. https://www.cellsignal.com/products/primary-antibodies/hamartin-tsc1-d43e2-rabbit-mab/6935. The antibody was validated by the manufacturer for western blotting and immunoprecipitation. Validation from our study: the antibody produces a band at the expected size on western blot with a decreased intensity in cells with TSC1 siRNA downregulation (Fig 2f).
Rabbit TFEB (Bethyl Laboratories, A303-673A), 110 citations. https://www.bethyl.com/product/A303-673A/TFEB+Antibody. The antibody was validated by the manufacturer for western blotting and immunoprecipitation. Validation from our study: the antibody produces a band at the expected size on the western blot with a decreased intensity in human cells with TFEB siRNA downregulation (Suppl Figure 4b) and in mouse cells with Tfeb shRNA downregulation (Figure 4i).
Rabbit TFEB (Cell Signaling Technology, 32361), 4 citations. https://www.cellsignal.com//products/primary-antibodies/tfeb-d4l2p-rabbit-mab/32361. The antibody was validated by the manufacturer for western blotting and immunoprecipitation. Validation from our study: the antibody produces a band at the expected size on the western blot.
Rabbit TFE3 (Cell Signaling Technology, 14779), 9 citations. https://www.cellsignal.com//products/primary-antibodies/tfe3-antibody/14779. The antibody was validated by the manufacturer for western blotting. Validation from our study: the antibody produces bands at the expected sizes on the western blot (Figure 3b,c,e,f).
Rabbit TFE3 (Millipore Sigma, HPA023881), 39 citations. https://www.sigmaaldrich.com/catalog/product/sigma/hpa023881?lang=en&region=US. The antibody was validated by the manufacturer for western blotting, immunofluorescence and immunohistochemistry. Validation from our study: the antibody gives the expected diffused cytoplasmic staining in wild type mouse cells (Suppl Fig 2b).
Rabbit Cathepsin K (Abcam, ab19027), 90 citations. https://www.abcam.com/cathepsin-k-antibody-ab19027.html. The antibody was validated by the manufacturer for western blotting. Validation from our study: the antibody produces a band at the expected size on the western blot.
Rabbit NPC1 (Abcam, ab134113), 23 citations. https://www.abcam.com/niemann-pick-c1-antibody-epr5209-ab134113.html. The antibody was validated by the manufacturer for western blotting, immunohistochemistry, immunofluorescence and flow cytometry. Validation from our study: the antibody produces a band at the expected size on the western blot and “punctate” cytoplasmic staining in immunohistochemistry.
Rabbit FLCN (Cell Signaling Technology, 3697), 22 citations. https://www.cellsignal.com/products/primary-antibodies/flcn-d14g9-rabbit-mab/3697?site-search-type=Products&N=4294956287&Ntt=flcn&fromPage=plp&_requestid=3910498. The antibody was validated by the manufacturer for western blotting and immunoprecipitation. Validation from our study: the antibody produces a band at the expected size on the western blot with a decreased intensity in cells with FLCN siRNA downregulation (Figure 5e and Suppl Figure 6c).
Chicken GFP (Abcam, ab13970), 2150 citations. https://www.abcam.com/gfp-antibody-ab13970.html. The antibody was validated by the manufacturer for western blotting and immunofluorescence. Validation from our study: the antibody produces the band at the expected size of the GFP-tagged protein.
Rabbit phosphorylated TFEB at serine 211 (S211) (Cell Signaling Technology, 37681), 3 citations. https://www.cellsignal.com//products/primary-antibodies/phospho-tfeb-ser211-e9s8n-rabbit-mab/37681. The antibody was validated by the manufacturer for western blotting. Validation from our study: the antibody produces a band at the expected size; band intensity correlated with cellular localization of TFEB.
Rabbit phosphorylated TFEB at serine 142 (S142) (Millipore Sigma, ABE1971). https://www.emdmillipore.com/US/en/product/Anti-phospho-TFEB-Ser142,MM_NF-ABE1971-I-25UL#overview. The antibody was validated by the manufacturer for western blotting. Validation from our study: the antibody produces a band at the expected size; band intensity correlated with cellular localization of

TFEB.
Rabbit RagC (Cell Signaling Technology, 9480), 8 citations. https://www.cellsignal.com//products/primary-antibodies/ragc-d8h5-rabbit-mab/9480. The antibody was validated by the manufacturer for western blotting, immunofluorescence, immunoprecipitation and flow cytometry. Validation from our study: the antibody produces a band at the expected size on the western blot.
Mouse Myc-tag (Cell Signaling Technology, 2276), 976 citations. https://www.cellsignal.com/products/primary-antibodies/myc-tag-9b11-mouse-mab/2276?site-search-type=Products&N=4294956287&Ntt=myc-tag&fromPage=plp. The antibody was validated by the manufacturer for western blotting, immunofluorescence, immunohistochemistry, immunoprecipitation, flow cytometry and chromatin immunoprecipitation. Validation from our study: the antibody produces a band at the expected size of the Myc-tagged protein on the western blot.
Rabbit HA-tag (Cell Signaling Technology, 3724), 866 citations. https://www.cellsignal.com/products/primary-antibodies/ha-tag-c29f4-rabbit-mab/3724?site-search-type=Products&N=4294956287&Ntt=3724s&fromPage=plp&_requestid=3918881. The antibody was validated by the manufacturer for western blotting, immunofluorescence, immunohistochemistry, immunoprecipitation, flow cytometry and chromatin immunoprecipitation. Validation from our study: the antibody produces a band at the expected size of the HA-tagged protein on the western blot.
Rabbit GPNMB (Cell Signaling Technology, 38313). https://www.cellsignal.com//products/primary-antibodies/gpnmb-e4d7p-xp-rabbit-mab/38313. The antibody was validated by the manufacturer for western blotting, immunohistochemistry and immunoprecipitation. Validation from our study: the antibody produces a band at the expected size on the western blot.
Rabbit CREB (Cell Signaling Technology, 9197), 569 citations. https://www.cellsignal.com//products/primary-antibodies/creb-48h2-rabbit-mab/9197. The antibody was validated by the manufacturer for western blotting, immunofluorescence, immunohistochemistry, immunoprecipitation, flow cytometry and chromatin immunoprecipitation. Validation from our study: the antibody produces a band at the expected size on the western blot and is present exclusively in the nuclear fraction in fractionated lysates.
Rabbit GAPDH (Cell Signaling Technology, 5174), 2230 citations. https://www.cellsignal.com//products/primary-antibodies/gapdh-d16h11-xp-rabbit-mab/5174. The antibody was validated by the manufacturer for western blotting, immunofluorescence and immunohistochemistry. Validation from our study: the antibody produces a band at the expected size on the western blot and is present exclusively in the cytoplasmic fraction in fractionated cells.

Eukaryotic cell lines

Policy information about cell lines

Cell line source(s)	Tsc1+/+, Tsc1-/-, Tsc2+/+, Tsc2-/- mouse embryonic fibroblasts (MEFs) were provided by Dr. David Kwiatkowski at Brigham and Women's Hospital Boston, MA, US. HeLa cells and HEK293T cells were purchased from ATCC. Hela-TFEB-GFP cells were provided by Shawn Ferguson (Yale University).
Authentication	Knockout and knockdown cells were authenticated by WB or RT-PCR. Overexpression cells were authenticated by WB or RT-PCR. Cell lines purchased from ATCC were kept in culture using all the standard methods to avoid contamination with other cell lines and validated by morphological analysis.
Mycoplasma contamination	Cells were tested for mycoplasma on a regular basis (every 2-3 months) and were confirmed negative.
Commonly misidentified lines (See ICLAC register)	N/A

Animals and other organisms

Policy information about studies involving animals; ARRIVE guidelines recommended for reporting animal research

Laboratory animals	8-week old female NOD-scid IL2R gamma null mice for xenograft experiments were obtained from Taconic. 18 months old female AJ Tsc2+/- mice were used for electron microscopy of the kidneys.
Wild animals	No wild animals were used.
Field-collected samples	No field-collected samples were used.
Ethics oversight	Animal studies were approved by the Brigham and Women's Hospital Animal Care and Use Committee.

Note that full information on the approval of the study protocol must also be provided in the manuscript.
